# How Does Vaccine-Induced Immunity Compare to Infection-Acquired Immunity in the Dynamics of COVID-19?

**DOI:** 10.3390/pathogens14020179

**Published:** 2025-02-11

**Authors:** Indunil M. Hewage, Dylan Hull-Nye, Elissa J. Schwartz

**Affiliations:** 1 Department of Mathematics & Statistics, Washington State University, Pullman, WA 99164, USA; indunil.hewage@wsu.edu (I.M.H.); dylan.hull-nye@wsu.edu (D.H.-N.); 2 School of Biological Sciences, Washington State University, Pullman, WA 99164, USA

**Keywords:** COVID-19 vaccination, vaccine-induced immunity, infection-acquired immunity, waning immunity, vaccinated reproduction number, backward bifurcations, local and global sensitivity analysis

## Abstract

Five years into the COVID-19 pandemic, the availability of effective vaccines has substantially reduced new cases, hospitalizations, and mortality. However, the waning of immunity has been a topic of particular interest in relation to disease control. The objective of this study is to investigate the impact of the decline in vaccine-induced immunity ($\omega_{1}$) and infection-acquired immunity ($\omega_{2}$) on disease dynamics. For this purpose, we use a compartmental model with seven compartments that accounts for differential morbidity, vaccination, and waning immunity. A compartmental model divides a population into distinct groups depending on their disease status. The temporal changes in the compartments are represented through ordinary differential equations (ODEs). The model is mathematically analyzed to show that a backward bifurcation (i.e., a perverse outcome) may occur when the vaccinated reproduction number ($R_{v}$) is equal to unity. Both local and global sensitivity analysis on the reproduction number reveal that the vaccine efficacy, waning of vaccine-induced immunity, vaccine coverage rate, coefficients of transmissibility, and the recovery rate for mild infections are the most sensitive parameters. The global sensitivity analysis on the cumulative number of infections shows that $\omega_{1}$ and $\omega_{2}$ are both pivotal parameters, while $\omega_{2}$ has a higher influence. Simulations on infections and mortality suggest that the changes in $\omega_{2}$ result in dynamics that are more pronounced compared to the dynamics resulting from the changes in $\omega_{1}$, thus indicating the importance of the duration of infection-acquired immunity in disease spread.

## 1. Introduction

COVID-19 is a highly contagious viral disease caused by a novel type of coronavirus known as *severe acute respiratory syndrome coronavirus 2* (SARS-CoV-2) [1]. COVID-19 is mainly transmitted by the breathing of air carrying aerosol particles containing the SARS-CoV-2 virus or by the eyes, nose, or mouth having contact with such particles [1]. Symptoms usually appear within two weeks after exposure to the virus, and can include a variety of conditions ranging from mild symptoms to severe illness and Long COVID (or Post-COVID conditions) [2]. After its first detection in Wuhan, China in December 2019, COVID-19 developed into a pandemic within the span of a year, and has currently become endemic in many countries [3]. As of 1 December 2024, the cumulative numbers of COVID-19 cases and disease-related deaths stand at 776,973,432 and 7,077,725, respectively [3].

Initial disease control and prevention efforts focused on the identification of infected individuals through aggressive testing and extensive contact tracing as well as on the reduction in exposure through the isolation of infected individuals, quarantine of exposed individuals, lockdowns, travel bans, social distancing, and the wearing of masks [1]. Later efforts focused on vaccination as a better strategy for disease control. There are currently several effective vaccines, with the Pfizer/BioNTech Comirnaty vaccine, Moderna COVID-19 vaccine, Oxford–AstraZeneca COVID-19 vaccine, Johnson & Johnson COVID-19 vaccine, Sinopharm COVID-19 vaccine, and the Sinovac-CoronaVac COVID-19 vaccine being some of the most administered vaccines in the world [3,4]. By the beginning of 2024, more than 13 billion COVID-19 vaccine doses have been administered worldwide [3].

While all existing COVID-19 vaccines are leaky (i.e., not completely effective at preventing infection), most of them are considerably efficacious against severe morbidity and mortality [5]. An independent analysis by the FDA based on the initial phase 3 clinical data for the Pfizer COVID-19 vaccine suggests that the vaccine can be 95% efficacious in preventing severe morbidity and mortality [6]. Some vaccines may only block infections, whereas others are protective against severe symptoms and death, but not infection or transmission [7,8,9]. For example, one study examines which of these two types of COVID-19 vaccines is more effective at a population level [9]. Despite the widespread accessibility of vaccines that are continually being enhanced, the control of SARS-CoV-2 infections has been particularly challenging due to reinfections and the emergence of new strains or variants [10,11]. Therefore, the waning of vaccine-induced immunity and infection-acquired immunity (i.e., natural immunity) are key factors that merit attention in a study focused on the epidemiological impact of vaccines.

COVID-19 is perhaps the most heavily studied disease of the recent past. The impressive corpus of mathematical modeling on COVID-19 is based on a variety of approaches, including continuous-time deterministic (ordinary differential equations (ODEs) and partial differential equations (PDEs)), discrete-time deterministic, statistical, and stochastic models [12,13,14,15,16,17,18,19,20,21,22,23,24,25,26]. Many of these studies have used compartmental models, where a population is categorized based on each individual’s disease status at a given moment [27]. While most studies employ a binary classification of symptomatic/asymptomatic infections [12,13,17,19,20,23], only a few account for differential morbidity based on the severity of symptoms [9,24,28,29]. A broader clinical spectrum of SARS-CoV-2 symptoms includes asymptomatic, presymptomatic, mild, moderate, severe, and critical infections [30].

In the mathematical modeling of epidemiology, the phenomenon of backward bifurcation entails dire epidemiological consequences. This undesirable outcome results from the disease-free equilibrium (i.e., steady state of the model free from infections) co-existing with two endemic equilibria (i.e., steady states of the model with persistent infections), one stable and the other unstable, when the reproduction number is less than unity [31,32,33]. The disease can become endemic even when $R_{0}<1$ (i.e., classical threshold), but is greater than a certain value $R_{0}^{*}$, which lies in the open interval (0,1). Thus, additional measures may be required to prevent the disease from becoming endemic or to bring $R_{0}$ below $R_{0}^{*}$ [32].

Several COVID-19 models exhibit the phenomenon of backward bifurcation [34,35,36,37,38]. For example, one ODE-based compartmental model accounts for the effects of vaccination and waning immunity on three sub-classes of infected individuals (i.e., symptomatic, asymptomatic, and hospitalized) [34]. A backward bifurcation in the model occurs in the context of low vaccination coverage and infection of vaccinated individuals. Moreover, an extended SVIR model of COVID-19 that categorizes the infected individuals as symptomatic or asymptomatic shows that a backward bifurcation can result from the coupling of the imperfect nature of the vaccine with the waning of natural immunity [37]. Another study uses distributed delay equations to examine a compartmental model for an endemic scenario of COVID-19, with decline in immunity from both vaccination and natural infection [35]. The direction of the bifurcation at the unity of the reproduction number of this model depends on the immunity waning rates.

Another COVID-19 model follows an extended version of the SVEIR structure to represent disease endemicity as well as waning immunity, and categorizes the infected individuals as presymptomatic infectious and symptomatic infectious [38]. This model also undergoes the phenomenon of backward bifurcation, which can be eliminated by increasing vaccine efficacy or decreasing disease transmissibility. The study shows that the waning of vaccine-induced immunity has a more pronounced effect on disease dynamics than does the waning of post-recovery immunity [38].

All the above studies exemplify the importance of waning immunity on disease dynamics and control. However, none of those studies seems to investigate the dynamics of subsequent disease waves. While at least one latter wave appears in the epidemic trajectories depicted in two works [35,37], these studies do not discuss the implications of the successive infection waves. Different studies have explored COVID-19 disease waves, but using tools such as time-varying transmission rates [39], stochastic models [40], and statistical data analysis [41].

An epidemiological model for an emerging infection by Hewage et al. [28] comprises seven compartments that account for vaccination and differential morbidity. The distinct compartments encompass susceptible, vaccinated, exposed, mildly symptomatic, moderately symptomatic, severely symptomatic, and recovered individuals. The model examines the impact of vaccine hesitancy on disease burden by means of a functional form for vaccine coverage, a feature not used in other studies. This model, however, does not account for the waning of natural or vaccine-induced immunity, an important factor in disease dynamics [22,35,37,38].

In this study, we build upon the between-host model in a previous study [28] to understand the dynamics of COVID-19 in the presence of imperfect vaccination and the waning of both vaccine-induced immunity and infection-acquired immunity. The incorporation of waning immunity allows us to examine the effects of recurring infections. We also include three infected classes, namely mild, moderate, and severe cases, to understand how differential morbidity affects the epidemic trajectories. The primary aims of our study are presented in terms of the following research questions:

1. What are the implications of the bifurcation at the unity of the reproduction number?
2. How sensitive are the model parameters to the vaccinated reproduction number; cumulative infections; first peak of infections; and the time until the first peak?
3. How do the waning of vaccine-induced immunity and the waning of infection-acquired immunity affect the disease dynamics within the population? Which factor has the higher impact on disease dynamics?
4. How efficacious of a vaccine is required to balance the effects of waning immunity?

We perform a comprehensive mathematical investigation that includes some preliminary results on the model’s qualitative nature, the derivation of equilibria and vaccinated reproduction number, and a bifurcation analysis. Then, we conduct both a local and global sensitivity analysis to understand how the input parameters influence the uncertainty of some model outputs. We also investigate the herd immunity threshold for the disease and how the two types of waning immunity affect the temporal variations of the time-dependent effective reproduction number. We next perform computer simulations on the model to explore how the two types of waning immunity affect the course of an epidemic and subsequent infection waves. Lastly, we quantify the level of vaccine effectiveness required to compensate for the two types of waning immunity.

The organization of this paper follows: Section 1 provides a review of the literature with a background to the current study. Section 2 elaborates the development of the model. A mathematical analysis of the model is provided in Appendix A. We present the results of the sensitivity analyses and numerical investigations in Section 3. The conclusions of the study are outlined in Section 4.

## 2. Model Development

The current study examines the dynamics of COVID-19 infections through an ODE-based compartmental model. Our model classifies a population into seven mutually exclusive compartments (with no overlaps), depending on each individual’s disease status at time *t*: unvaccinated susceptible (*S*); vaccinated susceptible (*V*); exposed (*E*); infected and experiencing mild symptoms ($I_{1}$); infected and experiencing moderate symptoms ($I_{2}$); infected and experiencing severe symptoms ($I_{3}$); and recovered (*R*). This model is based on supplementing the epidemiological model in a previous study [28] to include the waning of vaccine-induced immunity and infection-acquired immunity. We also exclude disease mortality from the compartment of moderately symptomatic individuals in light of COVID-19 disease dynamics [1,42]. Additionally, we assume that the vaccine coverage rate remains constant over time.

**Figure 1:**
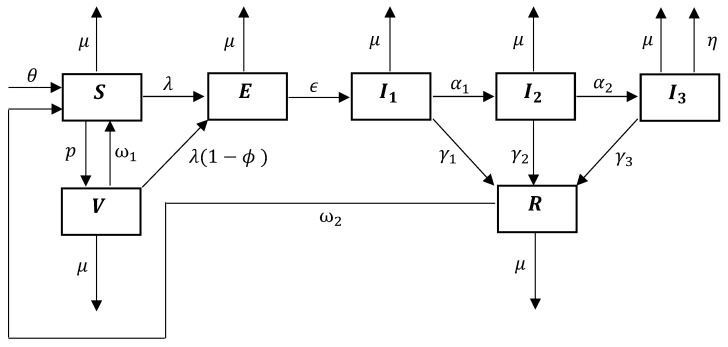
Schematic diagram for the dynamics of COVID-19 in the presence of vaccination and waning immunity.

Figure 1 schematically illustrates the transmission of COVID-19 in a population. The set of ordinary differential equations that mathematically represents the disease dynamics within the community is given in System (1). These ODEs are formulated based on the flow of individuals into and out of the seven compartments and the overall community over time:

(1) $$\begin{array}{c} \begin{array}{cc} \; & \frac{dS}{dt}=\theta +\omega_{1}V+\omega_{2}R-\left(\mu +p+\lambda \right)S;\\ \; & \frac{dV}{dt}=pS-\left[\mu +\omega_{1}+\lambda \left(1-\phi \right)\right]V\\ \; & \frac{dE}{dt}=\lambda S+\lambda \left(1-\phi \right)V-\left(\mu +\epsilon \right)E;\\ \; & \frac{dI_{1}}{dt}=\epsilon E-\left(\mu +\alpha_{1}+\gamma_{1}\right)I_{1};\\ \; & \frac{dI_{2}}{dt}=\alpha_{1}I_{1}-\left(\mu +\alpha_{2}+\gamma_{2}\right)I_{2};\\ \; & \frac{dI_{3}}{dt}=\alpha_{2}I_{2}-\left(\mu +\eta +\gamma_{3}\right)I_{3};\\ \; & \frac{dR}{dt}=\gamma_{1}I_{1}+\gamma_{2}I_{2}+\gamma_{3}I_{3}-\left(\mu +\omega_{2}\right)R. \end{array} \end{array}$$

The current model consists of 16 parameters as defined in Table 1. For the convenience of subsequent mathematical analysis, we use the bulk parameters $L_{1}=\mu +\alpha_{1}+\gamma_{1}$, $L_{2}=\mu +\alpha_{2}+\gamma_{2}$, and $L_{3}=\mu +\eta +\gamma_{3}$ to represent the total loss of the infected compartments $I_{1},I_{2},$ and $I_{3}$, respectively. The main assumptions used in formulating the model are provided below.

- Demographics: The per capita natural community mortality rate is μ. New individuals are recruited into the community at the rate θ=μN(0), where $N\left(t\right)=S\left(t\right)+V\left(t\right)+E\left(t\right)+I_{1}\left(t\right)+I_{2}\left(t\right)+I_{3}\left(t\right)+R\left(t\right)$. However, the population is not closed because of disease-induced deaths.
- Vaccination: Susceptible individuals are vaccinated at the rate *p*. The vaccine is both leaky and waning, but not all-or-nothing (i.e., providing no protection to a certain fraction of the vaccinated individuals and perfect lifelong protection to others [43]). The vaccine offers an individual some protection against infection, but not against severe disease or death in the event of an infection. The level of protection that the vaccine provides against infection is ϕ, which is defined as the probability of a vaccinated individual being successfully protected from infection. The vaccine-induced immunity wears off at the rate $\omega_{1}$.
- Force of infection and incidence rate: The time-varying and density-dependent (per capita) force of infection of the model is given by $\lambda \left(t\right)=\beta_{1}I_{1}\left(t\right)+\beta_{2}I_{2}\left(t\right)+\beta_{3}I_{3}\left(t\right)$. Unvaccinated susceptible and vaccinated susceptible individuals become exposed at the rates λ and (1−ϕ)λ, respectively. Thus, the disease incidence rate is given by λ[S+(1−ϕ)V].
- Disease transmission and progression: There is no vertical transmission of disease in the community. Disease transmission occurs by mass action that results from interactions between susceptible and infected individuals. The unvaccinated and the vaccinated (with a leaky protection) may acquire an infection. The disease incubation period is $\frac{1}{\epsilon }$. Individuals sequentially progress from mild symptoms through to severe symptoms (i.e., $I_{1}\to I_{2}\to I_{3}$). Individuals progress from $I_{1}$ to $I_{2}$ at the rate $\alpha_{1}$, and from $I_{2}$ to $I_{3}$ at the rate $\alpha_{2}$.
- Differential morbidity: The more severe the symptoms, the less likely it is that an individual will mingle with the community and will be able to transmit the disease (i.e., $\beta_{1}>\beta_{2}>\beta_{3}$). It takes longer to recover from more severe infections (i.e., $1/\gamma_{1}<1/\gamma_{2}<1/\gamma_{3}$). Only the individuals with severe symptoms succumb to the disease (at the rate η).
- Recovery and natural immunity: Individuals in $I_{1}$, $I_{2}$, and $I_{3}$ recover at the rates $\gamma_{1}$, $\gamma_{2}$, and $\gamma_{3}$, respectively. Recovered individuals transition to the same recovered compartment (*R*), irrespective of the severity of previous morbidity. Recovery from an infection does not provide permanent immunity. Infection-acquired immunity wanes at the rate $\omega_{2}$; the waning of natural immunity causes the recovered individuals to become susceptible again. This immunity does not depend on the severity of previous morbidity. Moreover, since the vaccine is effective merely against blocking an infection, only the infection-acquired immunity takes effect upon recovery, regardless of the vaccination status of individuals.

The units and values of the parameters used in the numerical investigations are presented in Table 2.

We refer the reader to Appendix A for an analytical investigation of Model (1). Appendix A.1 explores the qualitative nature of Model (1), including the existence and uniqueness of solutions, non-negativity and boundedness of solutions, and the positive invariance of the solution space. Appendix A.2 derives the disease-free equilibrium of Model (1) as well as the two important epidemiological metrics, namely the basic reproduction number ($R_{0}$, the reproduction number of the model in the absence of vaccination) and the vaccinated reproduction number ($R_{v}$, the reproduction number of the model in the presence of vaccination), along with some related results. Appendix A.3 analyzes the endemic equilibria of Model (1) and the direction of the bifurcation at $R_{v}=1$ (i.e., whether it is forward or backward). The model undergoes a backward bifurcation at $R_{v}=1$, given that the parameters satisfy the inequality in Equation (A9). This condition is further examined with respect to some key factors, including the vaccination parameters and immunity waning rates.

## 3. Numerical Results

In Section 3.1, we investigate both the local sensitivity and global sensitivity of some model outputs with respect to the model parameters. Numerical simulations on the vaccinated reproduction number, effective reproduction number, prevalence of infections, and disease mortality are provided in Section 3.2. Section 3.3 outlines the quantification of results in relation to vaccine efficacy and immunity waning rates.

### 3.1. Sensitivity Analysis of the Model

Here, we use both local and global sensitivity analysis methods to examine the sensitivity of Model (1) to changes in its parameters. Local sensitivity analysis is helpful in assessing the sensitivity of a model output in the neighborhood of a specific parameter space [51]. Conversely, global sensitivity analysis investigates the sensitivity of a model output to variations in parameters in their entire ranges, thus providing more comprehensive insights [52].

Section 3.1.1 explores the sensitivity of $R_{v}$ with respect to the model parameters using the direct sensitivity analysis method that is based on partial derivatives. In Section 3.1.2, we use the Partial Rank Correlation Coefficient (PRCC) method in tandem with Latin Hypercube Sampling (LHS) to examine the global sensitivity of $R_{v}$, cumulative infections over 1500 days, first peak of $I_{1}$, and the time until the first peak of $I_{1}$ to changes in the model parameters.

#### 3.1.1. Differential Sensitivity Analysis

Local sensitivity analysis aims to analyze the impact of small variations in the input parameters on a specific model output [51]. Here, we use the direct method (i.e., differential sensitivity analysis) to examine the local sensitivity of $R_{v}$ to changes in its parameter space over a small neighborhood.

Given a model parameter ρ, the formula for the normalized forward sensitivity index of $R_{v}$ with respect to ρ is given by $S_{\rho }=\frac{\rho }{R_{v}}\frac{\partial R_{v}}{\partial \rho }$ [53]. The sensitivity index $S_{\rho }$ provides valuable insights into the percentage change in $R_{v}$, which results from a small percentage change in the parameter ρ. Specifically, $S_{\rho }$ has a biological interpretation: a 1% change in the parameter ρ causes $R_{v}$ to change by $S_{\rho }$%. If $S_{\rho }>0$, then decreasing the value of ρ lowers $R_{v}$. If $S_{\rho }<0$, then increasing the value of ρ lowers $R_{v}$.

**Figure 2:**
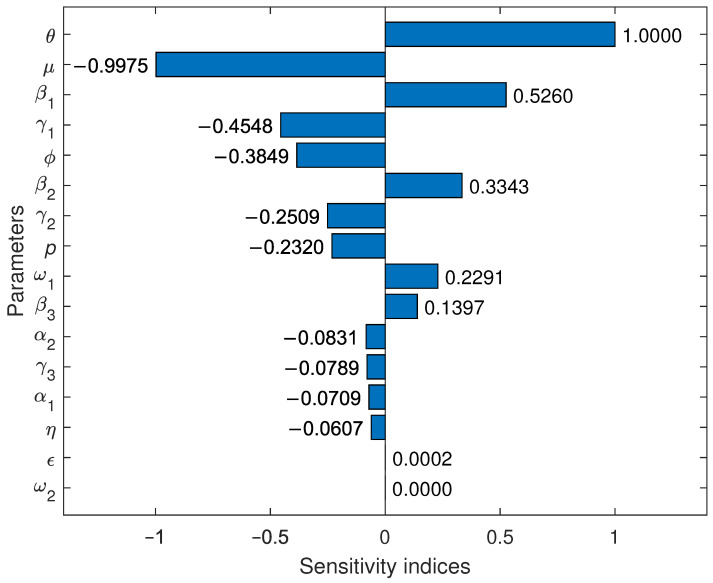
Sensitivity indices of the parameters in Model (1) with respect to $R_{v}$. The vertical axis denotes the parameters and the horizontal axis represents the sensitivity indices of those parameters.

The bar plot in Figure 2 illustrates the local sensitivity indices of the model parameters with respect to $R_{v}$. For example, $S_{\beta_{1}}=0.5260$ means that a 1% increase (or decrease) in the value of $\beta_{1}$ causes a 0.526% increase (or decrease) in $R_{v}$. Since $R_{v}$ is independent of $\omega_{2}$, we have $S_{\omega_{2}}=0$. The most negatively sensitive parameters are $\mu ,\gamma_{1},$ and ϕ, whereas the most positively sensitive parameters are $\theta ,\beta_{1}$, and $\beta_{2}$. At a moderate level, the parameters $\gamma_{2}$ and *p* are negatively sensitive, while $\omega_{1}$ and $\beta_{3}$ are positively sensitive.

$R_{v}$ is much less sensitive to the variations in the parameters $\epsilon ,\eta ,\alpha_{1},\gamma_{3},$ and $\alpha_{2}$. Changes in the incubation rate (ϵ) usually have a very mild impact on $R_{v}$. However, it is somewhat surprising that changes in the disease progression rates ($\alpha_{1}$ and $\alpha_{2}$) have a minor impact on $R_{v}$. Even though μ and θ are the most sensitive, as demographic parameters, these are less likely to be receptive to interventions. Thus, the local sensitivity analysis shows that the vaccinated reproduction number can greatly be reduced with control measures that prioritize $\phi ,\gamma_{1},\beta_{1}$, and $\beta_{2}$. Therefore, some effective interventions include enhancing the vaccine efficacy, increasing the recovery rates for mildly symptomatic individuals, and decreasing disease transmissibility.

#### 3.1.2. Partial Rank Correlation Coefficient (PRCC) Sensitivity Analysis

Local sensitivity analysis provides only limited insights into the sensitivity of model outputs because of its inability to explore the variations in the parameters over all admissible ranges. As such, PRCC sensitivity analysis is an efficient approach to global sensitivity analysis based on rank correlations. We use the Latin hypercube sampling (LHS) method to sample the parameters for the PRCC approach. The LHS method is a stratified sampling procedure without replacement that allows us to survey the whole parameter space. A total of 1000 samples are drawn from the ranges given in Table 2 as uniform probability distributions between the minimum value of the range (min) and the maximum value of the range (max).

We next calculate various model outputs (such as the reproduction number and the peak of infections) for each of the generated sample sets of parameters. We assess the strength and direction of the relationships between each model output and the parameters using PRCC values as sensitivity indices. The PRCC method works by comparing the ranks of model parameters with the ranks of the model outputs [54]. PRCC values vary between −1 and 1; a positive PRCC value indicates a positive correlation between the relevant parameter and the model output, whereas a negative PRCC value indicates a negative correlation [54]. The closer the PRCC value is to 1 or −1, the more that parameter contributes monotonically to changes in the output.

Here, we use the PRCC method to perform a global sensitivity analysis of the model parameters to $R_{v}$; the sum of the cumulative number of infections across mild ($I_{1}$), moderate ($I_{2}$), and severe ($I_{3}$) cases over the first 1500 days; the number of infections at the first peak, summed across the three infected classes $I_{1},I_{2}$, and $I_{3}$; and the sum of the times to $I_{1}^{\max},I_{2}^{\max}$, and $I_{3}^{\max}$. The monotonicity of the relationships was verified, as required for the PRCC analysis.

**Figure 3:**
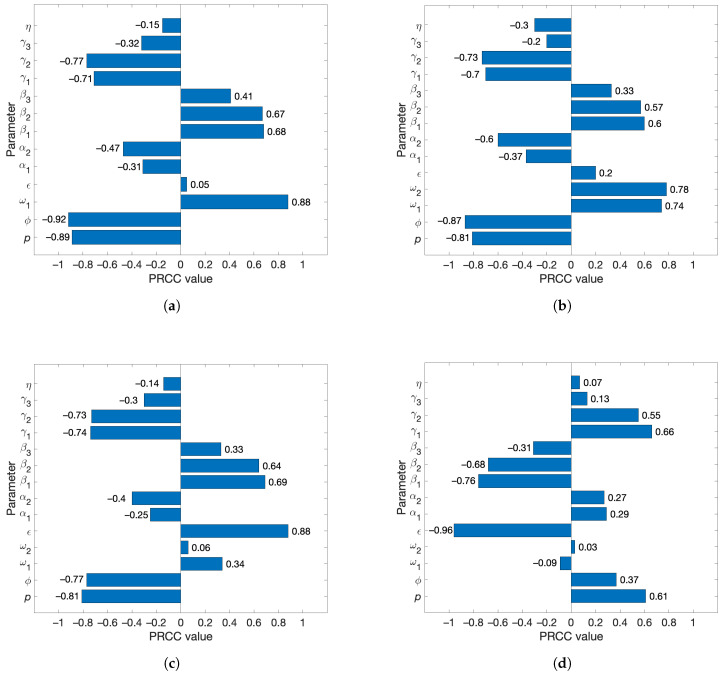
PRCC values of the model parameters (Table 2) with respect to four different model outputs. (**a**) Vaccinated reproduction number ($R_{v}$). (**b**) Sum of the cumulative number of mild, moderate, and severe cases over the first 1500 days. (**c**) Number of infections at the first peak, summed across the three infected classes ($I_{1}^{\max}+I_{2}^{\max}+I_{3}^{\max}$). (**d**) Sum of the times to $I_{1}^{\max},I_{2}^{\max}$, and $I_{3}^{\max}$.

As depicted in Figure 3a, the most influential parameters to the vaccinated reproduction number are the vaccine efficacy (ϕ), vaccine coverage rate (*p*), and the vaccine-induced immunity waning rate ($\omega_{1}$). The first two transmission parameters ($\beta_{1},\beta_{2}$) and the first two recovery parameters ($\gamma_{1},\gamma_{2}$) are also influential. It is expected that any reproduction number will be sensitive to transmission and recovery parameters. Intuitively, the vaccine efficacy and coverage rate are strongly negatively correlated with $R_{v}$; a more efficacious vaccine and a higher coverage rate should slow the spread of disease. The rate of waning of vaccine-induced immunity is strongly positively correlated with $R_{v}$. This indicates that the faster the vaccine-induced immunity wanes, the faster the disease spreads.

The equation for $R_{v}$ does not include the disease-acquired immunity waning rate ($\omega_{2}$), and hence $\omega_{2}$ does not appear in the PRCC plot for $R_{v}$. This, however, does not mean that $\omega_{2}$ has no effect on the dynamics of the disease. In order to discover its effects, we next examine the overall disease burden, measured as the sum of the cumulative number of infections across $I_{1}$, $I_{2}$, and $I_{3}$ over the first 1500 days. In Figure 3b, both waning rates ($\omega_{1}$ and $\omega_{2}$) show a nearly equal and high effect on this disease burden metric. Numerical simulations suggest that the infection curves undergo at least two waves over the course of the first 1500 days due to the effects of waning immunity. Thus, the high negative sensitivity indices for $\omega_{1}$ and $\omega_{2}$ here are reasonable. Moreover, the PRCC value of $\omega_{2}$ is slightly higher than that of $\omega_{1}$, indicating that the infection-acquired immunity waning has a higher influence on the cumulative infections than does the vaccine-induced immunity waning.

In the trajectories of infections across most parameter samples, the infected classes peak twice, and in some cases, there are three or four peaks. However, the first peak is always the highest. In Figure 3c,d, we show that the vaccine efficacy and coverage rate (ϕ and *p*) are very influential to the first (and largest) peak of infections as well as to the time to this peak. The respective recovery and transmission parameters are sensitive as well. The disease incubation rate (ϵ) has the highest PRCC value. Neither waning rate is strongly influential to the peaks across the parameter ranges. Interestingly, the bars representing the PRCC values for the peak (Figure 3c) for the time to this peak (Figure 3d), indicating how the parameters that drive the peak higher also cause the trajectory to reach that peak sooner.

Overall, the global sensitivity analysis reveals that the vaccine-induced immunity waning rate, vaccine efficacy, and the vaccine coverage rate ($\omega_{1},\phi ,p$) drive the changes in the vaccinated reproduction number, whereas both waning rates, vaccine efficacy, and the vaccine coverage rate ($\omega_{1},\omega_{2},\phi ,p$) drive the changes in the cumulative number of infections. However, the disease incubation rate, transmission coefficients, and the recovery rates ($\epsilon ,\beta_{1},\beta_{2},\gamma_{1},\gamma_{2}$) have a large influence on the peak of infections and the time to this peak. On a comparison of the impact of vaccine-induced immunity waning ($\omega_{1}$) and infection-acquired immunity waning ($\omega_{2}$) on the model outputs, $\omega_{2}$ is more influential for the changes in the cumulative infections, whereas $\omega_{1}$ is more influential for the peak of infections. Thus, some efficient disease control measures include producing vaccines with higher efficacy and longer protection, increasing the coverage of vaccination programs, reducing disease transmission, and expediting the recovery of infected individuals.

### 3.2. Computer Simulations

We now conduct some numerical simulations on Model (1) to understand the disease dynamics of COVID-19 under the effects of vaccination and waning immunity in order to propose the key factors that should be taken into account when planning control measures. The software packages used for the numerical simulations of the model include MATLAB R2023b and Mathematica 14.1. The initial values of the state variables are given below.
*S**V**E*$I_{1}$$I_{2}$$I_{3}$*R*99999001000

The vaccinated reproduction number, evaluated at the baseline parameter values (Table 2), is $R_{v}=1.5>1$. This number indicates that an outbreak will occur. In addition, we obtain $R_{0}=2.1>1.5=R_{v}$; this inequality implies that vaccination will reduce the reproduction number.

The concept of *herd immunity* refers to the herd effect or the indirect protection against a communicable disease that susceptible individuals receive when a sufficiently large number of individuals in the population is immune to the infection [55]. Thus, the herd immunity threshold is defined as the minimum proportion of the population that must be inoculated to reach the herd immunity level [55,56]. A narrative review of research on herd immunity in fighting COVID-19 is provided in the study by Suryawanshi et al. [57]. Moreover, in-depth, data-driven analyses of herd immunity in the context of COVID-19 can be found in several studies [58,59]. Specifically, Randolph et al. [58] discuss pertinent metrics for evaluating the societal cost of achieving global herd immunity against COVID-19. In addition, some studies emphasize the challenges in achieving herd immunity against COVID-19 even with mass vaccination [60,61].

The herd immunity threshold of a disease, denoted *H*, is computed by means of the formula $H=1-\frac{1}{R_{0}}$ [56]. A modified version, known as the critical vaccination level, is

(2) $$V_{c}=\frac{1-1/R_{0}}{\phi }=\frac{H}{\phi },$$

where ϕ is the effectiveness of the vaccine against transmission [55]. We have $V_{c}=H$ when the vaccine induces complete protection against infection (i.e., ϕ=1). For Model (1), we obtain $H=1-\frac{1}{2.1}=0.52$ and $V_{c}=\frac{0.52}{0.70}=0.74$. Thus, to attain herd immunity, a vaccination coverage of 52% is required for a flawless vaccine; a vaccination coverage of 74% is required for a vaccine with 70% efficacy. A drawback of this approach is that Equation (2) is a general formula that does not account for the specific dynamics of a model at hand, except the numerical value of its basic reproduction number. In particular, this formula fails to account for other important factors such as waning immunity.

**Figure 4:**
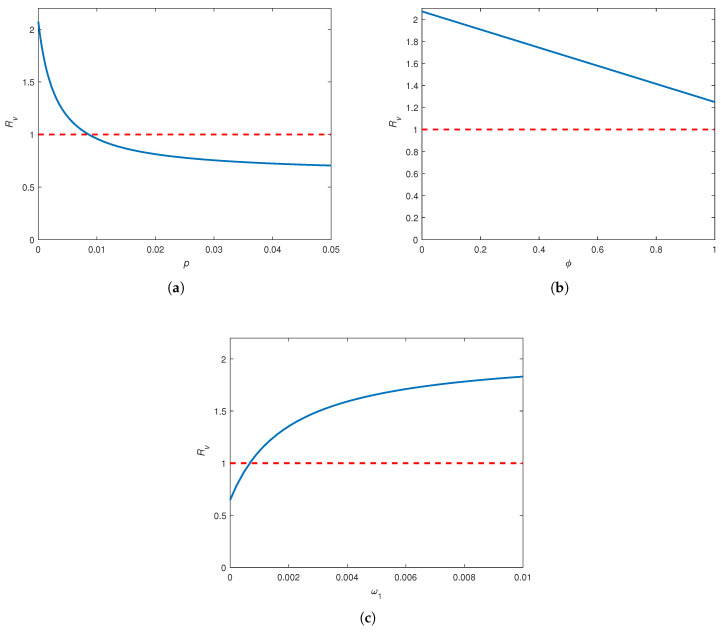
Variation in $R_{v}$ in accordance with vaccination parameters. The dashed line indicates the critical value of $R_{v}=1$. All parameters (except the one varied in each sub-figure) are fixed at the baseline values given in Table 2. (**a**) $R_{v}$ versus vaccine coverage rate (*p*). (**b**) $R_{v}$ versus vaccine efficacy (ϕ). (**c**) $R_{v}$ versus the waning rate for vaccine-induced immunity ($\omega_{1}$).

We now use numerical simulations to show how $R_{v}$ changes against the three vaccination parameters, vaccine coverage rate (*p*), vaccine efficacy (ϕ), and waning rate for vaccine-induced immunity ($\omega_{1}$). Figure 4a depicts that $R_{v}$ varies non-linearly with a substantial rational decay (resembling the decline observed in a hyperbolic rational function) when *p* increases over the given interval. This relationship indicates that $R_{v}$ drops sharply as *p* is increased up to 0.01. Specifically, the vaccination coverage rate should be greater than 0.01 in order to bring $R_{v}$ down from the critical level. Moreover, $R_{v}$ seems to level off at about 0.7 when p>0.05.

$R_{v}$ decreases linearly with ϕ at a considerably steep slope (Figure 4b). However, when the remaining parameters are fixed (Table 2), $R_{v}$ cannot be reduced below unity for any admissible value of ϕ. This means that even with a perfectly effective vaccine, an outbreak cannot be avoided. Figure 4c indicates that $R_{v}$ is an increasing function of $\omega_{1}$, but the slope of the curve decreases as $\omega_{1}$ increases. Thus, the reproduction number can be substantially decreased with vaccines that remain effective for at least 11 months (i.e., $\omega_{1}<0.003$, the point where the increase in $R_{v}$ seems to slow down). In particular, we need a vaccine with a protection that lasts longer than 2.7 years (i.e., $\omega_{1}<0.001$, the point that corresponds to $R_{v}=1$) to prevent an outbreak in the community. Further, $R_{v}$ appears to level off at around 1.8 for $\omega_{1}>0.01$.

**Figure 5:**
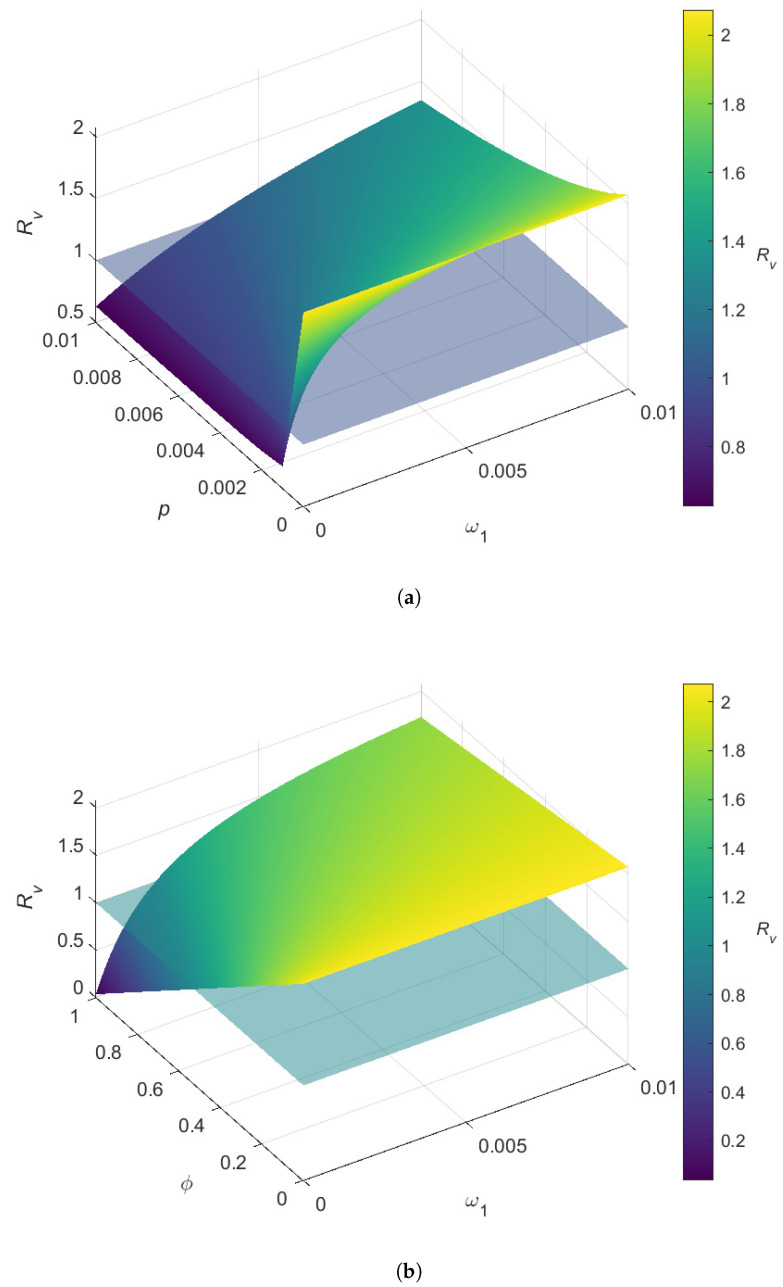
Three-dimensional plots of $R_{v}$ in accordance with the vaccine-related parameters. The transparent plane denotes the critical value of $R_{v}=1$. All parameters (except the pair of parameters varied in each sub-figure) are fixed at the baseline values given in Table 2. (**a**) Variation in $R_{v}$ in accordance with the vaccine coverage rate (*p*) and the waning rate for vaccine-induced immunity ($\omega_{1}$). (**b**) Variation in $R_{v}$ in accordance with the vaccine efficacy (ϕ) and the waning rate for vaccine-induced immunity ($\omega_{1}$).

The variations in $R_{v}$ with respect to pairs of vaccination parameters are depicted through the three-dimensional plots in Figure 5. Figure 5a shows that $R_{v}$ is significantly lower when the vaccine coverage rate takes larger values (p>0.002) and the waning rate for vaccine-induced immunity takes smaller values on the range $\omega_{1}<0.002$ (that corresponds to a vaccine lasting up to 1.37 years). Given a certain level for *p*, $R_{v}$ increases steadily as $\omega_{1}$ increases. If p<0.002, it is difficult to lower $R_{v}$ below 1, even if the vaccine-induced immunity does not wear off. Figure 5b indicates that given some level for ϕ, $R_{v}$ increases rapidly as $\omega_{1}$ increases. However, $R_{v}$ can be substantially reduced with higher values for the vaccine efficacy (ϕ) and lower values for the waning rate ($\omega_{1}$). Specifically, the requirements for decreasing $R_{v}$ below unity are approximately ϕ>0.55 and $\omega_{1}<0.002$.

The effective reproduction number ($R_{t}$) of a disease is a more pragmatic alternative to $R_{0}$ (or $R_{v}$) [62]. While $R_{0}$ gives the average number of secondary infections caused by an initial case in a completely susceptible population, $R_{t}$ estimates the expected number of secondary infections produced by an infectious case at any time point *t* during the course of an epidemic [63]. Thus, $R_{t}$ provides more insightful information than $R_{0}$ does in decision making while an epidemic is underway (when some individuals in the population are no longer susceptible). Several studies have estimated the effective reproduction number for COVID-19 using epidemic data [63,64,65]. $R_{t}$ is also defined as a data-driven measure of disease transmission that provides an estimate of the average number of new cases produced by each infectious individual on day *t* of an epidemic [66]. An interactive, continuously updated illustration of the monthly variations in $R_{t}$ and its 95% credible interval for the US and each state is provided on the webpage [66].

The effective reproduction number is formulated as $R_{t}=R_{0}\frac{S(t)}{N(t)}$, where $R_{0}$ is the basic reproduction number of the disease and $\frac{S(t)}{N(t)}$ is the proportion of susceptible individuals in the population at time *t* [62]. This formula can be revised based on the current model by changing S(t) to S(t)+(1−ϕ)V(t) to account for the vaccinated individuals who become susceptible due to vaccine failure. Hence, we obtain $R_{t}=R_{v}\frac{S(t)+(1-\phi )V(t)}{N(t)}$, which yields $\frac{R_{t}}{R_{v}}=\frac{S(t)+(1-\phi )V(t)}{N(t)}\leq 1$; this gives the relation $R_{t}\leq R_{v}$.

**Figure 6:**
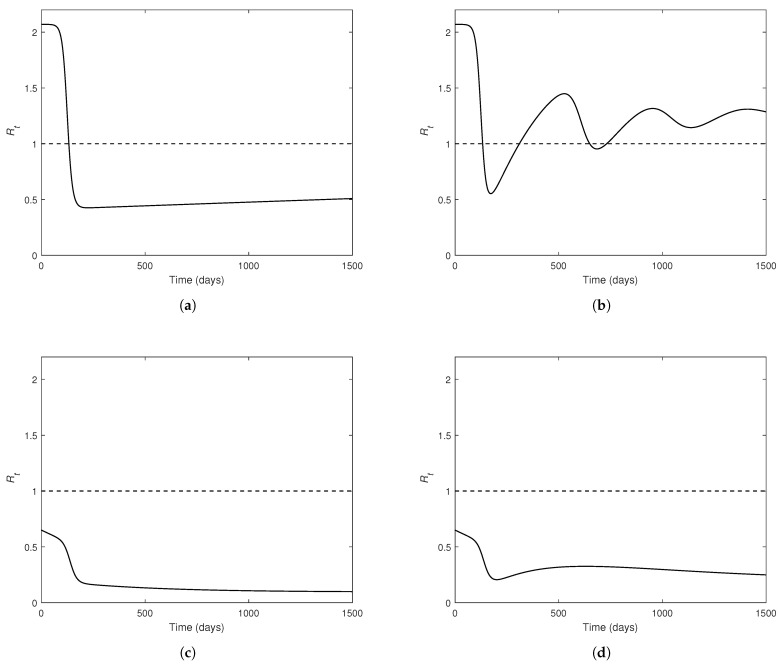

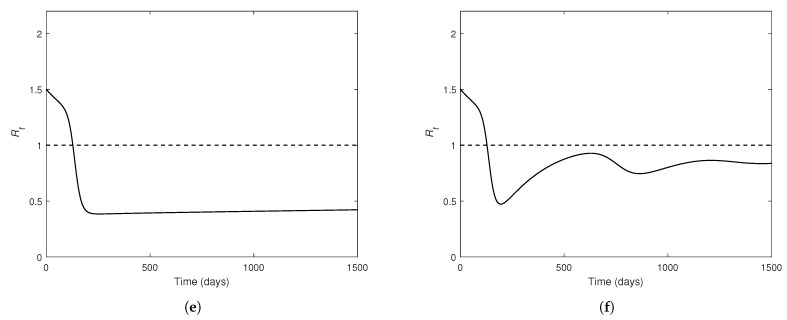
Variation in the effective reproduction number ($R_{t}$) over time, under different cases of the vaccine coverage rate (*p*), vaccine-induced immunity waning ($\omega_{1}$), and the infection-acquired immunity waning ($\omega_{2}$). All parameter values except those set to 0 are fixed as given in Table 2. The dashed line indicates the critical value of $R_{t}=1$. (**a**) No vaccination; no waning immunity (i.e., p=0, $\omega_{1}=0$, and $\omega_{2}=0$). (**b**) No vaccination; waning of infection-acquired immunity (i.e., p=0, $\omega_{1}=0$, and $\omega_{2}>0$). (**c**) With Vaccination; no waning of vaccine-induced immunity; no waning of infection-acquired immunity (i.e., p>0, $\omega_{1}=0$, and $\omega_{2}=0$). (**d**) With Vaccination; no waning of vaccine-induced immunity; waning of infection-acquired immunity (i.e., p>0, $\omega_{1}=0$, and $\omega_{2}>0$). (**e**) With Vaccination; waning of vaccine-induced immunity; no waning of infection-acquired immunity (i.e., p>0, $\omega_{1}>0$, and $\omega_{2}=0$). (**f**) With Vaccination; waning of vaccine-induced immunity; waning of infection-acquired immunity (i.e., p>0, $\omega_{1}>0$, and $\omega_{2}>0$).

Even though $R_{v}$ does not contain the waning rate for infection-acquired immunity ($\omega_{2}$), $R_{t}$ does, making $R_{t}$ an apt tool for exploring the effects of $\omega_{2}$ on the reproduction number. Figure 6 illustrates how $R_{t}$ changes over time for different cases based on the presence of vaccination as well as the waning of vaccine-induced and disease-acquired immunity.

A comparison of Figure 6a,c as well as Figure 6b,f illustrates how vaccination helps decrease the reproduction number. Figure 6b shows that $R_{t}$ undergoes some oscillations leading to several outbreaks over a period of 1500 days, when the disease-acquired immunity wanes in the absence of vaccination. However, there are fewer oscillations and no subsequent epidemics when the disease-acquired immunity wears off under the presence of vaccination, despite the decay of vaccine-induced immunity (Figure 6f).

An epidemic can be prevented and the oscillations can be greatly suppressed with a vaccine that provides long-lasting immunity (i.e., $\omega_{1}=0$), as seen in Figure 6c,d. Figure 6a,c,e show a disease spread that is greatly controlled, when the recovery from an infection provides lasting immunity (i.e., $\omega_{2}=0$). The consistent downward trend in Figure 6c shows that vaccination is highly effective in disease control when there is no waning of either type of immunity. Overall, the waning of infection-acquired immunity ($\omega_{2}$) has a higher influence on the oscillatory behavior of $R_{t}$ than does the waning of vaccine-induced immunity ($\omega_{1}$). In the inevitable event of natural immunity waning, vaccination (even with decaying protection) plays a pivotal role in mitigating the effective reproduction number (and hence disease burden).

**Figure 7:**
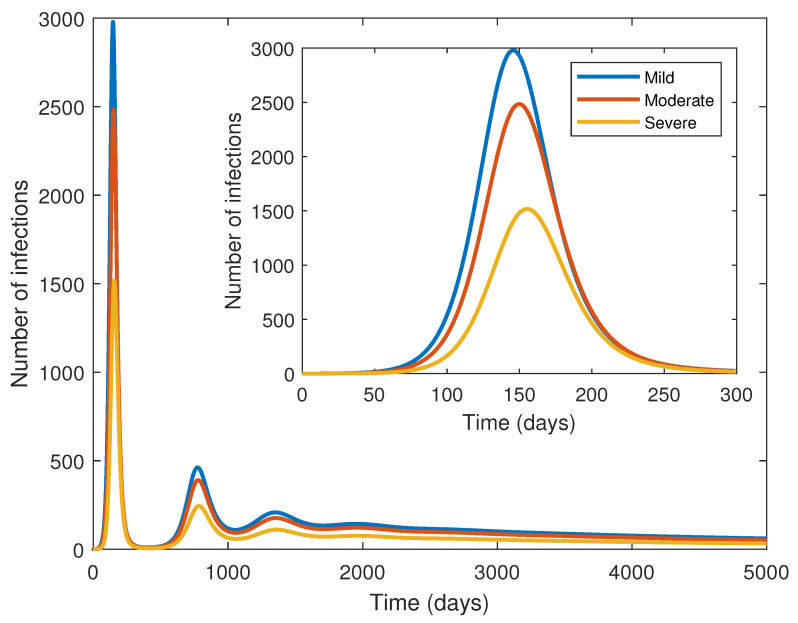
The number of cases with mild ($I_{1}$), moderate ($I_{2}$), and severe ($I_{3}$) infections across time. The inset provides a close-up view of the curves over a span of 300 days. All parameters are fixed at the baseline values given in Table 2.

Figure 7 tracks the changes in the number of infected individuals ($I_{1},I_{2}$, and $I_{3}$) over time. The disease propagation exhibits several waves over the course of 5000 days. The first peak of each curve is reached within 145–155 days. The peak for less intense infections is slightly earlier. At the first peak, the number of mild cases is about twice that of severe cases; there are approximately 67% more moderate cases than severe cases. These relative differences seem to remain consistent over the subsequent peaks. However, the latter waves are substantially flattened, and case numbers are also much lower during those waves. Eventually, the disease moves towards becoming endemic at lower case levels. The graph also confirms the absence of a periodic orbit (a solution curve that repeats itself over time) in the model.

**Figure 8:**
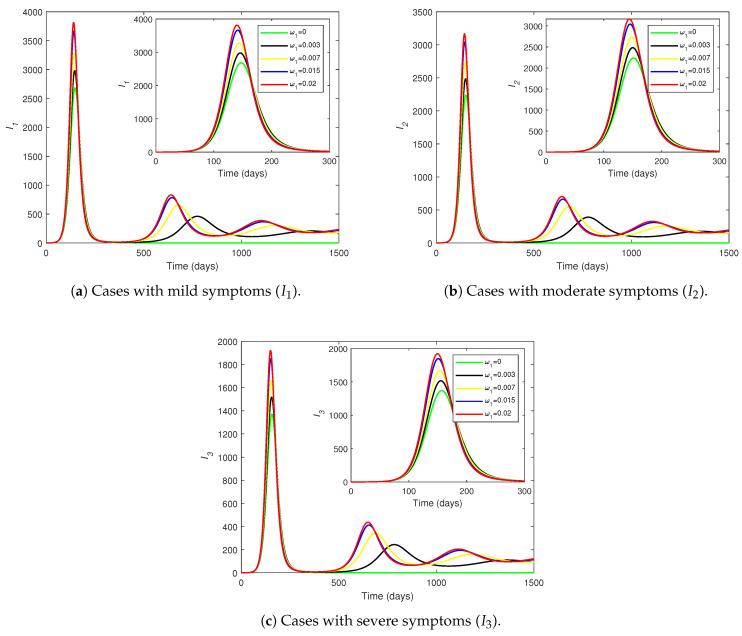
The changes in (**a**) $I_{1}$, (**b**) $I_{2}$, and (**c**) $I_{3}$ over time with various values for the waning of vaccine-induced immunity ($\omega_{1}$). The inset provides an enlarged view of the first wave of infections. All parameters (except $\omega_{1}$) are fixed at the baseline values given in Table 2. The vertical axes have different scales.

Figure 8 shows how the number of individuals in each infected compartment ($I_{1},I_{2},$ and $I_{3}$) evolves over time for different values of the waning of vaccine-induced immunity ($\omega_{1}$). The number of infections across all classes increases as $\omega_{1}$ increases. When $\omega_{1}=0$, there are no subsequent waves after the first peak. Thus, a non-waning vaccine resolves the infection after the first peak, but when the vaccine protection wears off, reinfections cause subsequent peaks. The number of cases at the first peak of each $I_{i}$ (for i=1,2,3) increases by approximately one-third as $\omega_{1}$ varies from 0 to 0.02.

For each level of morbidity, the time until the first peak is approximately the same for all displayed values of $\omega_{1}$, but the peak is delayed to a minuscule extent as $\omega_{1}$ decreases. However, the peaks for the subsequent waves are substantially delayed as $\omega_{1}$ decreases. This reveals that the faster waning of vaccine-induced protection causes the disease to peak earlier in the population. The relative drops in the peak number of infections (as $\omega_{1}$ decreases from 0.02 to 0) lessen as the symptoms become more lethal (Figure 8a–c), meaning that the peaks of mild (or moderate) infections drop more sharply than do the peaks of moderate (or severe) infections as the vaccine-induced immunity takes longer to wear off. The differences in the curves are negligible between the peak of the first wave and the beginning of the second wave, but the trajectories differ substantially thereafter.

**Figure 9:**
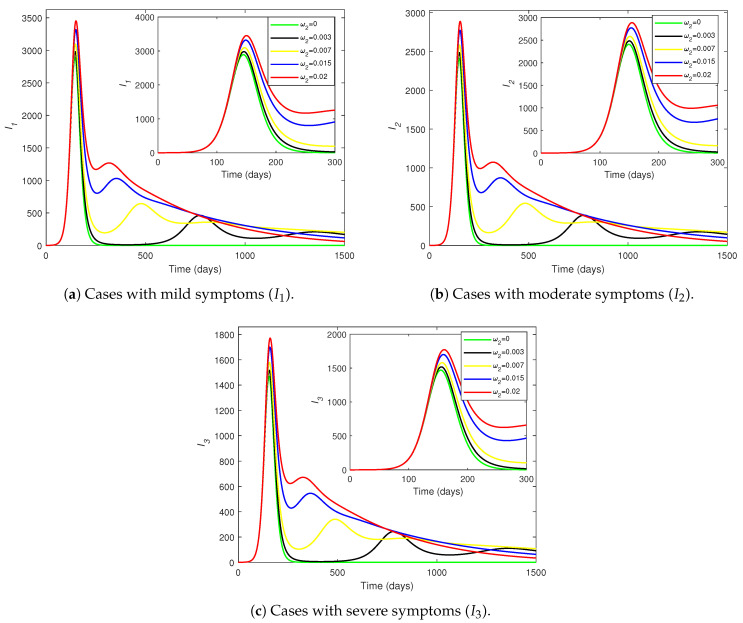
The changes in (**a**) $I_{1}$, (**b**) $I_{2}$, and (**c**) $I_{3}$ over time with various values for the waning of disease-acquired immunity ($\omega_{2}$). The inset provides an enlarged view of the first wave. All parameters (except $\omega_{2}$) are fixed at the baseline values given in Table 2. The vertical axes have different scales.

Figure 9 shows the variations in $I_{1},I_{2},$ and $I_{3}$ over time for different values of the waning rate for disease-acquired immunity ($\omega_{2}$). If $\omega_{2}=0$, the disease completely dies out after the first peak. Thus, no subsequent infection peaks occur in the absence of disease-acquired immunity waning. There are three discernible waves over the course of 1500 days, but for most values of $\omega_{2}$, the third peak is not reached within the displayed time period. For each level of morbidity, the curves for various values of $\omega_{2}$ fall on top of each other until the first peak, but the curves become considerably different afterwards. This observation for the first wave in Figure 9 contrasts with the corresponding observation for the first wave in Figure 8.

Given each $I_{i}$, the peak of the first wave delays very slightly as $\omega_{2}$ increases (contrary to the observations on the changes of $\omega_{1}$). However, the larger the value of $\omega_{2}$, the earlier the peaks of the subsequent waves are (in agreement with the changes of $\omega_{1}$). Larger values of $\omega_{2}$ correspond to higher peaks in each wave. Thus, the disease burden increases greatly as the infection-acquired immunity wanes faster. The graphs in Figure 8 and Figure 9 reiterate that the infection trajectories undergo several waves, with each wave being less severe than the one before.

A comparison of Figure 8 and Figure 9 suggests that $\omega_{2}$ has a greater impact on the continuation of infections than does $\omega_{1}$. The curves for $\omega_{1}$ in Figure 8 drop to almost 0 at the end of the first wave, whereas the curves for $\omega_{2}$ in Figure 9 drop only to about two-thirds of the peaks (for higher values of $\omega_{2}$) before rising again. The second peaks of the curves for $\omega_{2}$ (Figure 9) occur earlier than those of the curves for $\omega_{1}$ (Figure 8). The number of cases at the first peak of the curves for $\omega_{2}$ (Figure 9) vary in smaller ranges for each $I_{i}$ compared to the number of cases at the first peak of the curves for $\omega_{1}$ (Figure 8). Moreover, for larger waning rates, the peaks of the curves for $\omega_{1}$ (Figure 8) are higher than the peaks of the curves for $\omega_{2}$ (Figure 9) across all three infections, meaning that the vaccine-induced immunity waning ($\omega_{1}$) has a higher influence on the first wave of infections than does the infection-acquired immunity waning ($\omega_{2}$).

There are no subsequent waves after the first wave in either of the cases $\omega_{1}=0$ and $\omega_{2}=0.003$ or $\omega_{1}=0.003$ and $\omega_{2}=0$. A separate numerical simulation suggests that when $\omega_{2}=0$, there are no subsequent waves for any value of $\omega_{1}$; but, when $\omega_{1}=0$, subsequent waves do occur for $\omega_{2}>0.0045$. Thus, the main driver of the infection waves is the waning of disease-acquired immunity ($\omega_{2}$). As Figure 8 and Figure 9 indicate, infection-acquired immunity waning ($\omega_{2}$) can lead to substantially large subsequent waves, when coupled with the vaccine-induced immunity waning ($\omega_{1}$). Therefore, the waning of infection-acquired immunity has a higher negative impact on the infection dynamics than does the waning of vaccine-induced immunity.

**Figure 10:**
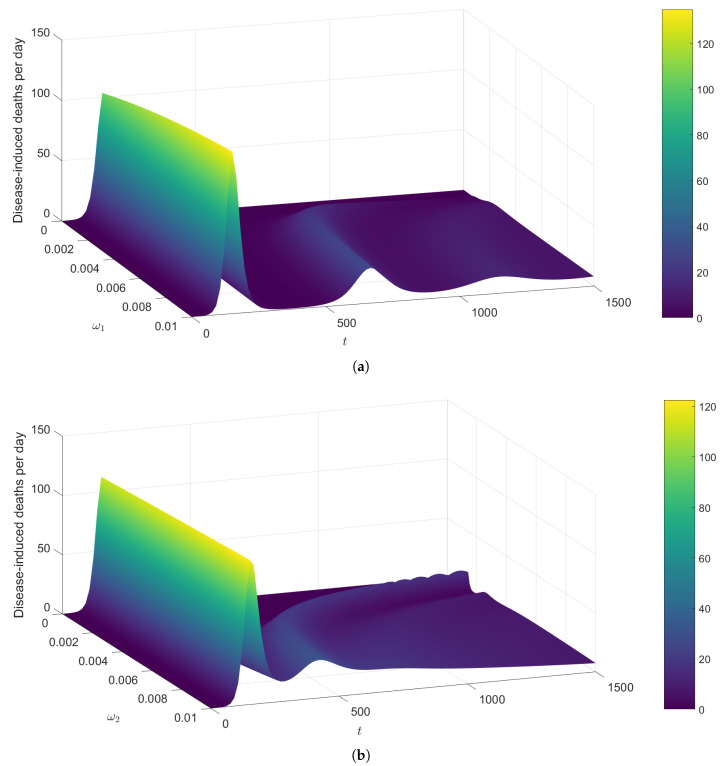
Changes in the number of disease-induced deaths per day (given by $\eta I_{3}$) over time (in days) and waning immunity. All parameters (except the one varied in each sub-figure) are fixed at the baseline values given in Table 2. The color scale indicates the number of deaths per day. (**a**) Variation in the disease-induced deaths per day in accordance with the waning rate for vaccine-induced immunity ($\omega_{1}$). (**b**) Variation in the disease-induced deaths per day in accordance with the waning rate for infection-acquired immunity ($\omega_{2}$).

We next examine the variation in the disease-induced deaths per day over time with respect to $\omega_{1}$ and $\omega_{2}$, using the term $\eta I_{3}$. Figure 10a illustrates at least three waves for the surface plot for higher values of vaccine-induced immunity waning ($\omega_{1}$). However, only one peak occurs when $\omega_{1}=0$, suggesting that a vaccine with a non-waning immunity can largely control case fatalities. At the first peak of the surface plot that occurs after approximately 140 days, the number of deaths per day stands at about 110 for all displayed values of $\omega_{1}$. The value of the peak increases almost imperceptibly as $\omega_{1}$ increases. Moreover, the number of deaths per day at the subsequent peaks remains well below 40.

In Figure 10b, lower values of infection-acquired immunity waning ($\omega_{2}$) give rise to more peaks over the time period displayed. There is only one peak for the surface when $\omega_{2}=0$, implying that the disease dies out after the first wave in the event that the infection-acquired immunity is permanent. The surface plot forms an erratic shape when t>500 and $0.002<\omega_{2}<0.006$. The first peak occurs at approximately 150 days, and the peak seems to slightly decrease as $\omega_{2}$ increases (contrary to the observations on $\omega_{1}$). Moreover, the dynamics of Figure 8 and Figure 10a as well as the dynamics of Figure 9 and Figure 10b seem comparable.

There are several waves of infections and deaths in the population in the presence of the waning of both types of immunity. The primary driving force of these waves is the waning of infection-acquired immunity. Sufficiently large levels of infection-acquired immunity waning can lead to the continuation of infections, even when the vaccine-induced protection is non-waning. However, the waning of vaccine-induced immunity has a larger impact on the peak of the first wave of infections than does the waning of infection-acquired immunity.

### 3.3. Quantification of Results on Waning Immunity

This section provides a quantification of the effects on disease burden that result from the waning of vaccine-induced immunity ($\omega_{1}$) and infection-acquired immunity ($\omega_{2}$). Here, we intend to answer the research question of how efficacious of a vaccine is required to reduce the population level impact of waning immunity.

To this end, we approximate the efficacy of a vaccine (ϕ) required to reduce the peaks of the first wave of infections across all three classes ($I_{1}$, $I_{2}$, $I_{3}$) by 40% (as compared to the reductions in those peaks with no immunity waning). First, we calibrate the value of vaccine efficacy (ϕ) that yields a 40% reduction in $I_{i}^{\max}$ under different levels of $\omega_{1}$, where $I_{i}^{\max}$ denotes the peak of the infected compartment $I_{i}$ (see Table 3).

For a given value of $\omega_{1}$, the vaccine efficacy required to decrease the peak of $I_{i}$ increases as i=1,2,3 increases (i.e., symptoms become more lethal). Given each $I_{i}$, larger efficacy levels are required to lower the peak as $\omega_{1}$ increases. Specifically, if the vaccine-induced immunity wanes after 6.67 months, then ϕ=0.89 is required to reduce the peak of mild infections by 40%. However, the efficacy level needed to achieve the same goal is much lower (ϕ=0.73), when the vaccine-induced protection lasts for at least 33.33 months.

We next calibrate the vaccine efficacy (ϕ) required to realize a 40% drop in $I_{i}^{\max}$ for each i=1,2,3 under three different values of infection-acquired immunity waning ($\omega_{2}$), namely 0.001, 0.003, and 0.005. The resulting quantifications are presented in Table 4. Given some value for $\omega_{2}$, the vaccine efficacy required to attain the above goal slightly increases as the symptoms become more severe (i.e., *i* increases over i=1,2,3 for $I_{i}$). Moreover, higher levels of waning necessitate higher efficacies to reduce the peak of infections.

If the infection-acquired immunity takes about 33.33 months to wane, then a vaccine with ϕ=0.81 is sufficient to reduce the peak of each infected class by 40%. However, if the immunity wanes faster (within 11.11 months), a vaccine with ϕ=0.83 is required to cut off the peak of all infections by 40%. When the time for waning is even shorter (within 6.67 months), the efficacy of the vaccine must be increased up to ϕ=0.85 to achieve the same goal. Interestingly, the relative increases in ϕ required in the case of increasing $\omega_{2}$ are lower than those required in the case of increasing $\omega_{1}$.

**Figure 11:**
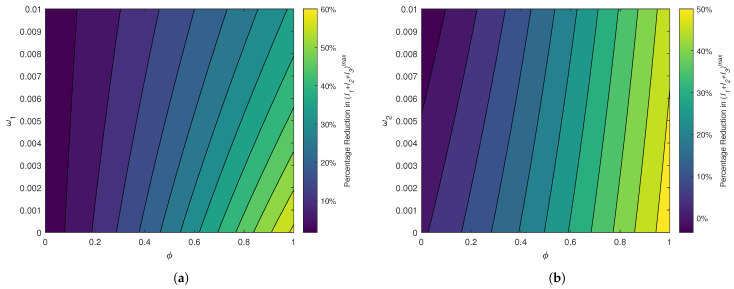
A heat map of the percentage reduction in the first peak of all three infected classes $I_{1}+I_{2}+I_{3}$ (as opposed to the case with no vaccination) in accordance with vaccine efficacy and waning immunity. All parameters (except the pair of parameters varied in each sub-figure) are fixed at the baseline values given in Table 2. (**a**) Percentage reduction in ${\left(I_{1}+I_{2}+I_{3}\right)}^{\max}$ with respect to the vaccine efficacy (ϕ) and the waning rate for vaccine-induced immunity ($\omega_{1}$). (**b**) Percentage reduction in ${\left(I_{1}+I_{2}+I_{3}\right)}^{\max}$ with respect to the vaccine efficacy (ϕ) and the waning rate for infection-acquired immunity ($\omega_{2}$).

Figure 11a depicts the percentage reduction in the peak of total infections with respect to the vaccine efficacy (ϕ) and vaccine-induced immunity waning ($\omega_{1}$). The peak infections can only be reduced by an optimal level of 60% even in the extreme case: ϕ≈1 and $\omega_{1}\approx 0$. Overall, ϕ>0.9 and $\omega_{1}<0.002$ are required to cut the peak of total infections by 50%. Figure 11b illustrates how the percentage reduction in the peak of total cases changes with respect to the vaccine efficacy (ϕ) and infection-acquired immunity waning ($\omega_{2}$). When the vaccine efficacy is less than 0.6, the reduction is no more than 20%, regardless of the value of $\omega_{2}$. However, ϕ>0.9 and $\omega_{2}<0.006$ can yield very high levels of reduction (by 50%) in the peak of infections. The variations in Figure 11a are starker than the variations in Figure 11b. For a given level of vaccine efficacy, the level of $\omega_{1}$ required to achieve a certain reduction in ${\left(I_{1}+I_{2}+I_{3}\right)}^{\max}$ is lower than the required level of $\omega_{2}$. Thus, longer vaccine-induced immunity is more important in reducing the peak of infections than longer infection-acquired immunity.

A comparison of Figure 11a,b as well as Table 3 and Table 4 indicates that the vaccine efficacy necessitated to reduce the first peak of infections by a certain percentage is higher for a given level of vaccine-induced immunity waning ($\omega_{1}$) than for the same level of disease-acquired immunity waning ($\omega_{2}$). Thus, the peaks of infections are more sensitive to the variations in $\omega_{1}$ (given a sufficiently large value for $\omega_{2}$) than to the variations in $\omega_{2}$ (given a sufficiently large value for $\omega_{1}$). Moreover, these results are consistent with the global sensitivity analysis (Figure 3c) as well as with the simulations on the first peaks of infection curves (Figure 8 and Figure 9). Thus, the disease burden, especially the overwhelming of healthcare facilities, can be reduced substantially with vaccines that provide long-lasting protection.

## 4. Conclusions

In this study, we investigated an extended SVEIR model that incorporates differential morbidity as well as the waning of both vaccine-induced immunity and infection-acquired immunity. The model was examined, both mathematically and numerically, to determine the direction of the bifurcation at the unity of the reproduction number, the sensitivity of the model parameters to some model outputs, how the waning of both types of immunity affects the disease dynamics within the community, and the efficacy of a vaccine that is required to eliminate the negative impact of waning immunity.

We first showed some preliminary mathematical results on the qualitative nature of Model (1). Then, we computed the disease-free equilibrium of the model and derived a quadratic equation that yields its endemic equilibria. The vaccinated reproduction number ($R_{v}$) of the model was computed by means of the NGM method. We performed a bifurcation analysis of the model using the sign of derivative method and the center manifold theory. Thus, we proved that the model exhibits the phenomenon of backward bifurcation at $R_{v}=1$ when it satisfies a certain inequality that consists of some model parameters. This perverse outcome disappears when the vaccine is perfect (ϕ=1) or when the disease-acquired immunity is permanent ($\omega_{2}=0$). Moreover, the waning of infection-acquired immunity plays a more prominent role in the occurrence of a backward bifurcation than does the waning of vaccine-induced immunity.

A local sensitivity analysis of the model through the differential sensitivity method revealed that the parameters that are most sensitive to $R_{v}$ are the vaccine efficacy (ϕ), transmission coefficients for infected individuals with mild and moderate symptoms ($\beta_{1}$ and $\beta_{2}$), and the recovery rate for infected individuals with mild symptoms ($\gamma_{1}$). The PRCC/LHS global sensitivity analysis showed that the vaccine efficacy (ϕ), vaccine coverage rate (*p*), and the waning rate for vaccine-induced immunity ($\omega_{1}$) are the most sensitive parameters to $R_{v}$. The results provided by the two methods are somewhat different because the differential sensitivity method examines the parameters one at a time in a local neighborhood of their point values, whereas the PRCC method examines all parameters simultaneously over their global ranges.

Moreover, the PRCC sensitivity analysis showed that both vaccine-induced immunity waning ($\omega_{1}$) and infection-acquired immunity waning ($\omega_{2}$) are highly sensitive to the cumulative number of infections; $\omega_{2}$ is more sensitive to this model output than $\omega_{1}$. Therefore, some effective ways to curtail an epidemic of COVID-19 include producing vaccines with high efficacy and long-lasting protection, increasing the coverage rate of vaccination programs, quarantining infected individuals, and promptly treating infected individuals with therapy that hastens their recovery.

Numerical simulations were performed to assess the impact of vaccination and waning immunity on $R_{v}$, the effective reproduction number ($R_{t}$), the levels of infections with mild, moderate, and severe symptoms, and the disease-induced deaths. We calculated the herd immunity threshold of the disease and showed that an overall vaccination coverage of 74% is required to achieve the herd effect. Simulations on the effective reproduction number over time showed that $\omega_{2}$ largely controls the temporal variations in $R_{t}$ as opposed to $\omega_{1}$. Specifically, the waning of immunity acquired from natural infection has a larger impact on maintaining $R_{t}$ below 1 than does the waning of immunity induced by a vaccine.

Figure 8, Figure 9, Figure 10 and Figure 11 demonstrated how infections and mortalities over time are much lower with smaller waning rates for vaccine-induced immunity and disease-acquired immunity. These simulations delineated that the waning of either immunity can lead to waves of disease spread over a long period of time. The faster the waning of either type of immunity, the larger the infection peaks become and the earlier the subsequent peaks occur. The quantification of results revealed the efficacy level that a vaccine must offer to reduce the peak of infections by 40% under different levels of vaccine-induced immunity waning ($\omega_{1}$) and infection-acquired immunity waning ($\omega_{2}$).

Overall, $\omega_{2}$ has a greater impact on the continuation of infections than does $\omega_{1}$. However, the variations in $\omega_{1}$ affect the first peak of infections more potently than do the variations in $\omega_{2}$. This result is consistent with the conclusions of a previous study [38], but that study does not examine subsequent infection waves. Hence, our study provides novel insights on the effects of the two types of waning immunity on overall disease dynamics.

There are several limitations to our study. The assumption that the progression of an infection takes the path $E\to I_{1}\to I_{2}\to I_{3}$ may not reflect the clinical progression of symptoms in general for all individuals in a population. Alternate paths may include $E\to I_{1}\to I_{3}$, $E\to I_{2}\to I_{3}$, or $E\to I_{3}$. In addition, we did not examine the role of vaccines in reducing the severity of symptoms because of the complexity that such a phenomenon would bring forth, especially in terms of the mathematical analysis. Furthermore, we did not discuss virus mutation and the generation of mutant variants in the current study. Future research may also benefit from incorporating a time-varying functional form for the vaccine coverage rate.

In conclusion, we used a COVID-19 epidemiological model to show that the waning of disease-induced immunity impacts the overall disease dynamics more prominently than does the waning of vaccine-induced immunity. The study also revealed that sufficiently efficacious vaccines, high vaccine coverage rates, and the slow waning of vaccine-induced immunity can substantially reduce infections and disease mortality. These results suggest some important directions that could guide a future study, especially by examining whether a program encouraging the uptake of booster shots and the vaccination of recovered individuals can help keep infections at bay.

## Figures and Tables

**Table 1:** Definitions of the parameters used in Model (1).

Parameter	Definition
μ	Natural mortality rate
θ	Recruitment rate
*p*	Vaccination coverage rate
ϕ	Vaccine efficacy
$\omega_{1}$	Rate of waning for vaccine-induced immunity
$\omega_{2}$	Rate of waning for infection-acquired immunity
ϵ	Incubation rate
$\alpha_{1}$	Rate of progression from mild to moderate symptoms
$\alpha_{2}$	Rate of progression from moderate to severe symptoms
$\beta_{1}$	Transmission coefficient for mildly symptomatic cases
$\beta_{2}$	Transmission coefficient for moderately symptomatic cases
$\beta_{3}$	Transmission coefficient for severely symptomatic cases
$\gamma_{1}$	Recovery rate for mildly symptomatic cases
$\gamma_{2}$	Recovery rate for moderately symptomatic cases
$\gamma_{3}$	Recovery rate for severely symptomatic cases
η	Diseased-induced death rate for severely symptomatic cases

**Table 2:** The units, baseline values, and ranges of the parameters used in the sensitivity analyses, computer simulations, and quantification of results for Model (1).

Parameter	Units	Baseline Value	Range = [Min, Max]	Source(s)
μ	$\frac{1}{\mathrm{day}}$	3.733 × 10^−5^	[3.192 × 10^−5^, 5.104 × 10^−5^] ^1^	[44]
θ	$\frac{\mathrm{individual}}{\mathrm{day}}$	3.733	[3.192, 5.104]	Set to μN(0) ^2^
*p*	$\frac{1}{\mathrm{day}}$	0.002	[0.002, 0.006]	Assumed ^3^
ϕ	dimensionless	0.700	[0.500, 0.950]	[45,46]
$\omega_{1}$	$\frac{1}{\mathrm{day}}$	0.003	[0.002, 0.006] ^4^	[47]
$\omega_{2}$	$\frac{1}{\mathrm{day}}$	0.003	[0.002, 0.006]	[47,48]
ϵ	$\frac{1}{\mathrm{day}}$	0.200	[0.071, 0.500] ^5^	[1,24]
$\alpha_{1}$	$\frac{1}{\mathrm{day}}$	0.200	[0.143, 0.333] ^6^	[24]
$\alpha_{2}$	$\frac{1}{\mathrm{day}}$	0.111	[0.071, 0.143] ^6^	Assumed ^6^
$\beta_{1}$	$\frac{1}{\mathrm{individual}\times \mathrm{day}}$	4.000 × 10^−6^	[3.500 × 10^−6^, 4.500 × 10^−6^]	[24]
$\beta_{2}$	$\frac{1}{\mathrm{individual}\times \mathrm{day}}$	3.000 × 10^−6^	[2.500 × 10^−6^, 3.500 × 10^−6^]	Assumed ^7^
$\beta_{3}$	$\frac{1}{\mathrm{individual}\times \mathrm{day}}$	2.000 × 10^−6^	[1.500 × 10^−6^, 2.500 × 10^−6^]	[24]
$\gamma_{1}$	$\frac{1}{\mathrm{day}}$	0.167	[0.100, 0.200] ^8^	[1,24]
$\gamma_{2}$	$\frac{1}{\mathrm{day}}$	0.125	[0.080, 0.150] ^8^	Assumed ^8^
$\gamma_{3}$	$\frac{1}{\mathrm{day}}$	0.100	[0.080, 0.150] ^8^	[1,24]
η	$\frac{1}{\mathrm{day}}$	0.077	[0.067, 0.100] ^9^	[24,49,50]

(1) Based on an average life span of 73.4 years, an average minimum life span of 53.68 years, and an average maximum life span of 85.83 years for both sexes. (2) N(0) = 100,000. (3) This value is highly dependent on the population, and hence may vary significantly from region to region. (4) Assumed based on an average protection period of 10 months and a range of 6–15 months. (5) Based on an average range of 2–14 days. (6) $\alpha_{1}$ assumed based on an average period of 5 days and a range of 3–7 days; $\alpha_{2}$ assumed based on an average period of 9 days and a range of 7–14 days in consideration of the inequality $\alpha_{1}>\alpha_{2}$. (7) Based on the relation $\beta_{1}>\beta_{2}>\beta_{3}$. (8) $\gamma_{1}$ chosen based on an average period of 6 days and a range of 5–10 days; $\gamma_{2}$ chosen based on an average period of 8 days and a range of 6.5–12.5 days; $\gamma_{3}$ chosen based on an average period of 10 days and a range of 6.5–12.5 days in consideration of the inequality $\gamma_{1}>\gamma_{2}>\gamma_{3}$. (9) Based on an average period of 13 days and a range of 10–15 days.

**Table 3:** Quantification of results on ϕ and $\omega_{1}$. All parameters (except ϕ and $\omega_{1}$) are fixed at the baseline values given in Table 2.

Waning of Vaccine-Induced Immunity ($\omega_{1}$)	Goal (Reducing the Peak of Infections)	Required Vaccine Efficacy (ϕ)
	Reduce $I_{1}^{\max}$ by 40%	ϕ=0.7321
0.001 (33.33 months)	Reduce $I_{2}^{\max}$ by 40%	ϕ=0.7409
	Reduce $I_{3}^{\max}$ by 40%	ϕ=0.7543
	Reduce $I_{1}^{\max}$ by 40%	ϕ=0.8071
0.003 (11.11 months)	Reduce $I_{2}^{\max}$ by 40%	ϕ=0.8167
	Reduce $I_{3}^{\max}$ by 40%	ϕ=0.8315
	Reduce $I_{1}^{\max}$ by 40%	ϕ=0.8851
0.005 (6.67 months)	Reduce $I_{2}^{\max}$ by 40%	ϕ=0.8959
	Reduce $I_{3}^{\max}$ by 40%	ϕ=0.9117

**Table 4:** Quantification of results on ϕ and $\omega_{2}$. All parameters (except ϕ and $\omega_{2}$) are fixed at the baseline values given in Table 2.

Waning of Infection-Acquired Immunity ($\omega_{2}$)	Goal (Reducing the Peak of Infections)	Required Vaccine Efficacy (ϕ)
	Reduce $I_{1}^{\max}$ by 40%	ϕ=0.7865
0.001 (33.33 months)	Reduce $I_{2}^{\max}$ by 40%	ϕ=0.7955
	Reduce $I_{3}^{\max}$ by 40%	ϕ=0.8100
	Reduce $I_{1}^{\max}$ by 40%	ϕ=0.8075
0.003 (11.11 months)	Reduce $I_{2}^{\max}$ by 40%	ϕ=0.8168
	Reduce $I_{3}^{\max}$ by 40%	ϕ=0.8318
	Reduce $I_{1}^{\max}$ by 40%	ϕ=0.8265
0.005 (6.67 months)	Reduce $I_{2}^{\max}$ by 40%	ϕ=0.8369
	Reduce $I_{3}^{\max}$ by 40%	ϕ=0.8525

## Data Availability

Data are contained within the article.

## Appendix A. Mathematical Analysis of the Model

### Appendix A.1. Preliminary Results

The current model consists of a system of seven coupled, non-linear, and autonomous ODEs that correspond to the seven state variables $S,\;V,\;E,\;I_{1},\;I_{2},\;I_{3},\;\mathrm{and}\;R$, all of which are functions of time. Furthermore, the model has the non-negative initial conditions

(A1) $$S\left(0\right)>0,\;V\left(0\right)\geq 0,\;E\left(0\right)\geq 0,\;I_{1}\left(0\right)\geq 0,\;I_{2}\left(0\right)\geq 0,\;I_{3}\left(0\right)\geq 0,\;R\left(0\right)\geq 0.$$

The local existence and uniqueness of solutions to System (1) are assured by the fact that the system is defined over a polynomial vector field. Hence, we analyze only the non-negativity and the boundedness of solutions as well as the positive invariance of the solution space in detail.

**Theorem A1.** 
*Given that the initial conditions are non-negative, solutions to System (1) are bounded and non-negative for all t≥0.*

**Proof.** To this end, we show that all components of System (1) remain inside the positive orthant for all t>0. Assume that S(0)>0, but there exists $t=t_{1}$ for which $S(t_{1})=0$. Suppose that $t_{1}=\inf T$, where T={t>0:S(t)<0,V(t),R(t)>0}. Then, $\frac{dS(t_{1})}{dt}\geq \theta +\omega_{1}V+\omega_{2}R>0$. This inequality implies that $S(t_{1}-\Delta t)<0$ for all sufficiently small values of Δt; a contradiction to $t_{1}=\inf T$. A similar argument can be constructed for S(0)=0, which yields S(t)≥0 for all t≥0. Thus, it follows that

$$\frac{dV}{dt}=pS-\left[\mu +\omega_{1}+\lambda \left(1-\phi \right)\right]V\geq -\left[\mu +\omega_{1}+\lambda \left(1-\phi \right)\right]V.$$

Solving this differential inequality yields

$$V\left(t\right)\geq V\left(0\right)\exp\left(-\left(\mu +\omega_{1}\right)t-\left(1-\phi \right)\int_{0}^{t}\left(\beta_{1}I_{1}\left(s\right)+\beta_{2}I_{2}\left(s\right)+\beta_{3}I_{3}\left(s\right)\right)ds\right)\geq 0.$$

Since S(t),V(t)≥0 for all t≥0, the third equation of System (1) gives

$$\frac{dE}{dt}=\lambda S+\lambda \left(1-\phi \right)V-\left(\mu +\epsilon \right)E\geq -\left(\mu +\epsilon \right)E.$$

The solution to this differential inequality is $E\left(t\right)\geq E\left(0\right)\exp\left(-(\mu +\epsilon )t\right)\geq 0$. Analogous arguments can be used to prove that $I_{1},I_{2},I_{3},$ and *R* also remain non-negative for all t≥0. To prove the boundedness of solutions, we sum up the seven equations of System (1) to obtain $\frac{dN}{dt}\leq \theta -\mu N-\eta I_{3}$; this inequality simplifies to $\frac{dN}{dt}\leq \theta -\mu N$ by the fact that $I_{3}>0$. Solving the simplified inequality yields $N\left(t\right)\leq \max\left\{N\left(0\right),\frac{\theta }{\mu }\right\}=\frac{\theta }{\mu }$. Hence, ${lim sup}_{t\to \infty }N\left(t\right)=\frac{\theta }{\mu }$. Thus, the boundedness of solutions to System (1) is established. □

**Theorem A2.** 
*The closed and bounded set*

$$\Omega =\left\{\left(S,V,E,I_{1},I_{2},I_{3},R\right)\in R_{+}^{7}:\;0<N\left(t\right)\leq \frac{\theta }{\mu }\right\}$$

*yields a positively invariant region for System (1).*

**Proof.** Suppose that $N\left(0\right)\leq \frac{\theta }{\mu }$. Thus, the initial condition given by Equation (A1) belongs to Ω. Moreover, from the proof of Theorem A1, it follows that $N\left(t\right)\leq \max\left\{N\left(0\right),\frac{\theta }{\mu }\right\}=\frac{\theta }{\mu }.$ Thus, the proof is completed. □

### Appendix A.2. Disease-Free Equilibrium and Reproduction Number

In epidemiological modeling, the disease-free equilibrium (DFE) is the steady state of an autonomous ODE system that corresponds to the infection-free status of a disease. The DFE of System (1) can be computed by setting all derivatives equal to zero along with $E=I_{1}=I_{2}=I_{3}=0$; doing so yields

(A2) $$E^{0}=\left(\frac{\theta (\mu +\omega_{1})}{\mu (\mu +\omega_{1}+p)},\frac{p\theta }{\mu (\mu +\omega_{1}+p)},0,0,0,0,0\right).$$

The parameter $\omega_{2}$ does not appear in $E^{0}$; this observation is also true for other disease-related parameters such as ϵ, η, $\alpha_{i}$s, $\beta_{i}$s, and $\gamma_{i}$s.

The basic reproduction number (denoted $R_{0}$) is an important metric in infectious disease modeling that measures the contagiousness of a pathogen. Mathematically, $R_{0}$ estimates the expected number of secondary cases generated by a single infected individual introduced into a completely susceptible population [62,67]. In more theoretical terms, $R_{0}\leq 1$ implies that the DFE is locally asymptotically stable, and $R_{0}>1$ implies that the DFE is unstable [53].

We formulate the reproduction number of Model (1) by means of the next generation matrix (NGM) method, attributed to both Diekmann et al. [68] and van den Driessche and Watmough [69]. Since a control strategy is in effect, we call the reproduction number in our model the *vaccinated reproduction number* and denote it as $R_{v}$. Thus, the standard definition of the basic reproduction number can be revised to interpret $R_{v}$ as the expected number of secondary infections generated by a primary infectious case, when introduced to a susceptible population where vaccination is readily accessible.

**Theorem A3.** 
*The vaccinated reproduction number of System (1) is given by*

(A3)
$$R_{v}=\frac{\theta \epsilon \left(\mu +\omega_{1}+\left(1-\phi \right)p\right)\left(L_{2}L_{3}\beta_{1}+L_{3}\beta_{2}\alpha_{1}+\beta_{3}\alpha_{1}\alpha_{2}\right)}{\mu \left(\mu +\epsilon \right)\left(\mu +\omega_{1}+p\right)L_{1}L_{2}L_{3}}.$$

**Proof.** The non-infected and infected compartments of System (1) are given by the vectors $X={\left(S,V,R\right)}^{T}$ and $Y={\left(E,I_{1},I_{2},I_{3}\right)}^{T}$, respectively. For the category *Y*, we have

$$\begin{array}{c} \frac{dY}{dt}={\left(\frac{dE}{dt},\frac{dI_{1}}{dt},\frac{dI_{2}}{dt},\frac{dI_{3}}{dt}\right)}^{⊺}=F-V, \end{array}$$

where

$$\begin{array}{c} F=\left(\begin{array}{c} \left(\beta_{1}I_{1}+\beta_{2}I_{2}+\beta_{3}I_{3}\right)S+\left(\beta_{1}I_{1}+\beta_{2}I_{2}+\beta_{3}I_{3}\right)\left(1-\phi \right)V\\ 0\\ 0\\ 0 \end{array}\right) \end{array}$$

and

$$\begin{array}{c} V=\left(\begin{array}{c} (\mu +\epsilon )E\\ -\epsilon E+L_{1}\;I_{1}\\ -\alpha_{1}I_{1}+L_{2}\;I_{2}\\ -\alpha_{2}I_{2}+L_{3}I_{3} \end{array}\right). \end{array}$$

F denotes all rates from *X* to *Y*; V denotes all remaining rates. We compute the Jacobians of F and V, and evaluate them at the DFE (Equation (A2)) to obtain

$$\begin{array}{c} F=\left(\begin{array}{cccc} 0 & \frac{\theta \beta_{1}\left(\mu +\omega_{1}+\left(1-\phi \right)p\right)}{\mu (\mu +\omega_{1}+p)} & \frac{\theta \beta_{2}\left(\mu +\omega_{1}+\left(1-\phi \right)p\right)}{\mu (\mu +\omega_{1}+p)} & \frac{\theta \beta_{3}\left(\mu +\omega_{1}+\left(1-\phi \right)p\right)}{\mu (\mu +\omega_{1}+p)}\\ 0 & 0 & 0 & 0\\ 0 & 0 & 0 & 0\\ 0 & 0 & 0 & 0 \end{array}\right) \end{array}$$

and

$$\begin{array}{c} V=\left(\begin{array}{cccc} \mu +\epsilon & 0 & 0 & 0\\ -\epsilon & L_{1} & 0 & 0\\ 0 & -\alpha_{1} & L_{2} & 0\\ 0 & 0 & -\alpha_{2} & L_{3} \end{array}\right). \end{array}$$

The matrix product $FV^{-1}$ is called the *next generation matrix*. Finally, the spectral radius of $FV^{-1}$ gives the vaccinated reproduction number

(A4) $$R_{v}=\rho \left(FV^{-1}\right)=\frac{\theta \epsilon \beta_{1}\left(\mu +\omega_{1}+\left(1-\phi \right)p\right)}{\mu \left(\mu +\epsilon \right)\left(\mu +\omega_{1}+p\right)L_{1}}+\frac{\theta \epsilon \beta_{2}\alpha_{1}\left(\mu +\omega_{1}+\left(1-\phi \right)p\right)}{\mu \left(\mu +\epsilon \right)\left(\mu +\omega_{1}+p\right)L_{1}L_{2}}+\frac{\theta \epsilon \beta_{3}\alpha_{1}\alpha_{2}\left(\mu +\omega_{1}+\left(1-\phi \right)p\right)}{\mu \left(\mu +\epsilon \right)\left(\mu +\omega_{1}+p\right)L_{1}L_{2}L_{3}},$$

which can be simplified to obtain Equation (A3). □

**Remark A1.** 
*$R_{v}$ is the expected number of secondary infections generated by an index case introduced into a susceptible population. In particular, the first, second, and third terms in Equation (A4), namely $\frac{\theta \epsilon \beta_{1}\left(\mu +\omega_{1}+\left(1-\phi \right)p\right)}{\mu \left(\mu +\epsilon \right)\left(\mu +\omega_{1}+p\right)L_{1}}$, $\frac{\theta \epsilon \beta_{2}\alpha_{1}\left(\mu +\omega_{1}+\left(1-\phi \right)p\right)}{\mu \left(\mu +\epsilon \right)\left(\mu +\omega_{1}+p\right)L_{1}L_{2}}$, and $\frac{\theta \epsilon \beta_{3}\alpha_{1}\alpha_{2}\left(\mu +\omega_{1}+\left(1-\phi \right)p\right)}{\mu \left(\mu +\epsilon \right)\left(\mu +\omega_{1}+p\right)L_{1}L_{2}L_{3}}$, represent the contributions to $R_{v}$ from the stages of mild ($I_{1}$); moderate ($I_{2}$); and severe ($I_{3}$) symptoms, respectively.*

**Remark A2.** 
*We can write the ratio $\frac{R_{v}}{R_{0}}=\frac{\mu +\omega_{1}+\left(1-\phi \right)p}{\mu +\omega_{1}+p}$, where $R_{0}=\frac{\theta \epsilon (L_{2}L_{3}\beta_{1}+L_{3}\beta_{2}\alpha_{1}+\beta_{3}\alpha_{1}\alpha_{2})}{\mu \left(\mu +\epsilon \right)L_{1}L_{2}L_{3}}$ is the basic reproduction number of Model (1) in the absence of vaccination. Since 0≤ϕ≤1, it follows that $\frac{R_{v}}{R_{0}}\leq 1\Rightarrow R_{v}\leq R_{0}$. This analytically proves that vaccination reduces the reproduction number.*

**Remark A3.** 
*$R_{v}$ does not comprise the parameter $\omega_{2}$, and it is the only model parameter that does not appear in $R_{v}$. Note that $\omega_{2}$ constitutes a path for new infections within the population. However, by definition, $R_{v}$ is defined for a population with a single infected individual; in this case, there are no recovered individuals to undergo recurring infections. The absence of the key parameter $\omega_{2}$ in $R_{v}$ indicates that the reproduction number does not yield a complete picture of disease dynamics in models with waning natural immunity [70]. Thus, the model may undergo a backward bifurcation at $R_{v}=1$.*

**Corollary A1.** 
*The disease-free equilibrium (A2) of Model (1) is locally asymptotically stable when $R_{v}<1,$ and it is unstable when $R_{v}>1$.*

**Proof.** The proof follows from the conclusions of Theorem 2 of the study by van den Driessche and Watmough [69]. Additionally, the proof of Theorem A4 serves as a proof of this corollary. □

### Appendix A.3. The Endemic Equilibria

The endemic equilibria of an epidemic model are the fixed points associated with the endemic state(s) of a disease. Thus, the infection variables typically assume non-zero values at endemic equilibria. We compute the endemic equilibria of System (1) by setting all its component equations to 0 and solving the resulting system of simultaneous algebraic equations for the state variables. Let $\Lambda_{1}=\frac{\epsilon (L_{2}L_{3}\beta_{1}+L_{3}\beta_{2}\alpha_{1}+\beta_{3}\alpha_{1}\alpha_{2})}{L_{1}L_{2}L_{3}}$ and $\Lambda_{2}=\frac{\epsilon (L_{2}L_{3}\gamma_{1}+L_{3}\gamma_{2}\alpha_{1}+\gamma_{3}\alpha_{1}\alpha_{2})}{L_{1}L_{2}L_{3}\left(\mu +\omega_{2}\right)}$. We have

(A5) $$\begin{array}{c} E^{1}=\left(S^{*},V^{*},E^{*},I_{1}^{*},I_{2}^{*},I_{3}^{*},R^{*}\right), \end{array}$$

where

(A6) $$\begin{array}{c} S^{*}=\frac{\left(\theta +\omega_{2}\Lambda_{2}E^{*}\right)\left(\mu +\omega_{1}+\left(1-\phi \right)\Lambda_{1}E^{*}\right)}{\left(\mu +\left(1-\phi \right)\Lambda_{1}E^{*}\right)\left(\mu +p+\Lambda_{1}E^{*}\right)+\omega_{1}\left(\mu +\Lambda_{1}E^{*}\right)},\;V^{*}=\frac{P(\theta +\omega_{2}\Lambda_{2}E^{*})}{\left(\mu +\left(1-\phi \right)\Lambda_{1}E^{*}\right)\left(\mu +p+\Lambda_{1}E^{*}\right)+\omega_{1}\left(\mu +\Lambda_{1}E^{*}\right)},\;\\ I_{1}^{*}=\frac{\epsilon }{L_{1}}E^{*},\;\;\;\;\;\;\;\;\;\;\;\;\;\;\;\;\;\;\;\;\;I_{2}^{*}=\frac{\alpha_{1}\epsilon }{L_{1}L_{2}}E^{*},\;\\ I_{3}^{*}=\frac{\alpha_{1}\alpha_{2}\epsilon }{L_{1}L_{2}L_{3}}E^{*},\;\;\;\;\;\;\;\;\;\;\;\;\;\;\;\;\;\;\;R^{*}=\Lambda_{2}E^{*}. \end{array}$$

Note that $E^{*}$ is given by the solution(s) of the quadratic equation

(A7) $$\begin{array}{c} aE^{2}+bE+c=0, \end{array}$$

where

(A8) $$\begin{array}{c} \begin{array}{cc} a & =\left(1-\phi \right)\Lambda_{1}^{2}\left(\mu +\epsilon -\omega_{2}\Lambda_{2}\right),\\ b & =\Lambda_{1}\left[\left(\mu +\epsilon \right)\left(\left(1-\phi \right)\left(\mu +p\right)+\mu +\omega_{1}\right)-\left(\theta \left(1-\phi \right)\Lambda_{1}+\left(\mu +\omega_{1}+p\left(1-\phi \right)\right)\omega_{2}\Lambda_{2}\right)\right],\;\mathrm{and}\\ c & =\left(\mu +p\right)\left(\mu +\epsilon \right)\left(\mu +\omega_{1}\right)-\theta \left(\mu +\omega_{1}+p\left(1-\phi \right)\right)\Lambda_{1}-p\omega_{1}\left(\mu +\epsilon \right)=\mu \left(\mu +\epsilon \right)\left(\mu +p+\omega_{1}\right)\left(1-R_{v}\right), \end{array} \end{array}$$

all of which are aggregate expressions containing the model parameters. However, only the positive (real) solutions of this quadratic equation are epidemiologically relevant.

### Appendix A.4. Bifurcation Analysis

In this section, we show that System (1) can exhibit a backward bifurcation at $R_{v}=1$ under a certain condition. The phenomenon of backward bifurcation causes a disease to become endemic even when the associated reproduction number is less than 1. First observed in 1992 by Huang et al. [71], backward bifurcations have been heavily explored in both within-host and between-host models [32,33].

A few methods can be used to determine the nature of the bifurcation of a model at $R_{0}=1$ [33]. First, we use the sign of derivative method [72] to derive an explicit condition for the occurrence of a backward bifurcation in Model (1). Then, we perform a normal form analysis based on the center manifold theory to prove that the system exhibits a backward bifurcation subject to a specific condition.

The sign of derivative method builds upon solving the model equations at equilibria to obtain an equation of the form F(I)=0, where *I* is an infection-related state variable in the system, usually the first infected variable at which infection is initiated [33]. The steady states are dependent on model parameters, but not on time. Suppose that ρ is a parameter that appears in the expression for $R_{v}$, such that $\frac{\partial R_{v}}{\partial \rho }>0$. Let $\rho =\rho^{*}$ when $R_{v}=1$. The model then undergoes a backward bifurcation at $R_{v}=1$, if ${\left.\frac{\partial I}{\partial \rho }\right|}_{I=0,\rho =\rho^{*}}<0$.

**Lemma A1.** 
*System (1) undergoes a backward bifurcation at $R_{v}=1$ if and only if*

(A9)
$$\begin{array}{c} \left(\mu +\epsilon -\omega_{2}\Lambda_{2}\right){\left(\mu +\omega_{1}+p\left(1-\phi \right)\right)}^{2}<\mu p\phi \left(1-\phi \right)\left(\mu +\epsilon \right). \end{array}$$

**Proof.** We begin by observing that we can leverage the quadratic equation for endemic equilibria (A7) for this purpose. Define F:[0,∞)↦R by $F\left(E\right)=aE^{2}+bE+c$, where a,b, and *c* are described as in Equation (A8). Treating *E* as a function of the model parameter θ yields F(E(θ),θ)=0. Observe that $\frac{\partial R_{v}}{\partial \theta }>0$. Let $\theta =\theta^{*}$ when $R_{v}=1$. We have

$$\frac{\partial F}{\partial \theta }=-\left(1-\phi \right)\Lambda_{1}^{2}E-\left(\mu +\omega_{1}+p\left(1-\phi \right)\right)\Lambda_{1}$$

and

$$\frac{\partial F}{\partial E}=2aE+b.$$

We then obtain

$$\frac{\partial E}{\partial \theta }=-\frac{\frac{\partial F}{\partial \theta }}{\frac{\partial F}{\partial E}}=\frac{\left(1-\phi \right)\Lambda_{1}^{2}E+\left(\mu +\omega_{1}+p\left(1-\phi \right)\right)\Lambda_{1}}{2aE+b}.$$

Evaluating the above partial derivative at E=0 and $\theta =\theta^{*}$ yields

$${\left.\frac{\partial E}{\partial \theta }\right|}_{E=0,\theta =\theta^{*}}=\frac{\left(\mu +\omega_{1}+p\left(1-\phi \right)\right)\Lambda_{1}}{b^{*}},$$

where $b^{*}$ is the value of *b* at $\theta =\theta^{*}$. Since the numerator is positive, the sign of this fraction depends on that of $b^{*}$. Specifically, ${\left.\frac{\partial E}{\partial \theta }\right|}_{E=0,\theta =\theta^{*}}<0$, if $b^{*}<0$. Hence, we obtain

$$\left(\mu +\epsilon \right)\left(\left(1-\phi \right)\left(\mu +p\right)+\mu +\omega_{1}\right)-\left(\theta^{*}\left(1-\phi \right)\Lambda_{1}+\left(\mu +\omega_{1}+p\left(1-\phi \right)\right)\omega_{2}\Lambda_{2}\right)<0,$$

which yields

$$\left(\mu +\epsilon \right)\left(\left(1-\phi \right)\left(\mu +p\right)+\mu +\omega_{1}\right)<\frac{\mu \left(\mu +\epsilon \right)\left(\mu +p+\omega_{1}\right)\left(1-\phi \right)}{\mu +\omega_{1}+\left(1-\phi \right)p}+\left(\mu +\omega_{1}+p\left(1-\phi \right)\right)\omega_{2}\Lambda_{2}.$$

Further algebraic simplifications give the more concise condition

$$\left(\mu +\epsilon -\omega_{2}\Lambda_{2}\right){\left(\mu +\omega_{1}+p\left(1-\phi \right)\right)}^{2}<\mu p\phi \left(1-\phi \right)\left(\mu +\epsilon \right).$$

□

Next, we use a center manifold analysis based on Theorem 4.1 of the study by Castillo-Chavez and Song [31] to determine the direction of the bifurcation at $R_{v}=1$.

**Theorem A4.** 
*If Condition (A9) holds, System (1) undergoes a backward bifurcation at $R_{v}=1$ with respect to $\beta_{1}$; this bifurcation can be characterized as follows:*

- *There are two endemic equilibria in a local neighborhood of $R_{v}=1$ such that $R_{v}\leq 1$. The DFE as well as the higher of the two endemic equilibria are locally asymptotically stable in this case.*
- *There exists a unique endemic equilibrium in a local neighborhood of $R_{v}=1$ such that $R_{v}>1$. The DFE is unstable and the endemic equilibrium is locally asymptotically stable in this case.*

**Proof.** We consider $\beta_{1}$ as the key parameter in the center manifold analysis. At $R_{v}=1$, we have $\beta_{1}^{*}=\frac{\mu \left(\mu +\epsilon \right)\left(\mu +p+\omega_{1}\right)L_{1}}{\theta \epsilon (\mu +\omega_{1}+\left(1-\phi \right)p)}-\frac{\beta_{2}\alpha_{1}}{L_{2}}-\frac{\beta_{3}\alpha_{1}\alpha_{2}}{L_{2}L_{3}}$. We represent System (1) concisely using the equation

$$\frac{dx}{dt}=f\left(x,\beta_{1}\right),\;f:R^{7}\times R,\;f\in C^{2}\left(R^{7}\times R\right),$$

where $x=(S,V,E,I_{1},I_{2},I_{3},R)$ and $f(E^{0},\beta_{1})=0$ for all values of $\beta_{1}$. The Jacobian matrix of System (1) evaluated at the DFE (A2) and at $\beta_{1}=\beta_{1}^{*}$ is given by

$$J\left(E^{0},\beta_{1}^{*}\right)=\left(\begin{array}{ccccccc} -(\mu +p) & \omega_{1} & 0 & -\frac{\theta \left(\mu +\omega_{1}\right)\beta_{1}^{*}}{\mu (\mu +\omega_{1}+p)} & -\frac{\theta \left(\mu +\omega_{1}\right)\beta_{2}}{\mu (\mu +\omega_{1}+p)} & -\frac{\theta \left(\mu +\omega_{1}\right)\beta_{3}}{\mu (\mu +\omega_{1}+p)} & \omega_{2}\\ p & -(\mu +\omega_{1}) & 0 & -\frac{p\theta \left(1-\phi \right)\beta_{1}^{*}}{\mu (\mu +\omega_{1}+p)} & -\frac{p\theta \left(1-\phi \right)\beta_{2}}{\mu (\mu +\omega_{1}+p)} & -\frac{p\theta \left(1-\phi \right)\beta_{3}}{\mu (\mu +\omega_{1}+p)} & 0\\ 0 & 0 & -(\mu +\epsilon ) & \frac{\theta \left(\mu +\omega_{1}+\left(1-\phi \right)p\right)\beta_{1}^{*}}{\mu (\mu +\omega_{1}+p)} & \frac{\theta \left(\mu +\omega_{1}+\left(1-\phi \right)p\right)\beta_{2}}{\mu (\mu +\omega_{1}+p)} & \frac{\theta \left(\mu +\omega_{1}+\left(1-\phi \right)p\right)\beta_{3}}{\mu (\mu +\omega_{1}+p)} & 0\\ 0 & 0 & \epsilon & -L_{1} & 0 & 0 & 0\\ 0 & 0 & 0 & \alpha_{1} & -L_{2} & 0 & 0\\ 0 & 0 & 0 & 0 & \alpha_{2} & -L_{3} & 0\\ 0 & 0 & 0 & \gamma_{1} & \gamma_{2} & \gamma_{3} & -(\mu +\omega_{2}) \end{array}\right).$$

$J\left(E^{0},\beta_{1}^{*}\right)$ has a single zero eigenvalue, whereas all other eigenvalues consist of a negative real part. The left and right eigenvectors of the Jacobian matrix corresponding to the zero eigenvalue are given by

$$\begin{array}{cc} v & ={\left(\begin{array}{c} 0\\ 0\\ 1\\ \frac{\mu +\epsilon }{\epsilon }\\ \frac{J_{\left(3,5\right)}L_{3}+J_{\left(3,6\right)}\alpha_{2}}{L_{2}L_{3}}\\ \frac{J_{\left(3,6\right)}}{L_{3}}\\ 0 \end{array}\right)}^{⊺}\mathrm{and}\;w=\left(\begin{array}{c} -\frac{\omega_{1}K_{1}+\left(\mu +\omega_{1}\right)K_{2}}{\mu (\mu +p+\omega_{1})}\\ -\frac{\left(\mu +p\right)K_{1}+pK_{2}}{\mu (\mu +p+\omega_{1})}\\ 1\\ \frac{\epsilon }{L_{1}}\\ \frac{\epsilon \alpha_{1}}{L_{1}L_{2}}\\ \frac{\epsilon \alpha_{1}\alpha_{2}}{L_{1}L_{2}L_{3}}\\ \Lambda_{2} \end{array}\right), \end{array}$$

where $K_{1}=\frac{p(1-\phi )(\mu +\epsilon )}{\mu +\omega_{1}+p\left(1-\phi \right)}$ and $K_{2}=\frac{\left(\mu +\omega_{1}\right)\left(\mu +\epsilon \right)}{\mu +\omega_{1}+p\left(1-\phi \right)}-\omega_{2}\Lambda_{2}$. We then construct the sums

$$a=\sum_{k,i,j=1}^{7}v_{k}w_{i}w_{j}\frac{\partial^{2}f_{k}}{\partial x_{i}\partial x_{j}}\;\;\mathrm{and}\;\;b=\sum_{k,i=1}^{7}v_{k}w_{i}\frac{\partial^{2}f_{k}}{\partial x_{i}\partial \beta_{1}},$$

where $f_{1},\ldots ,f_{7}$ are the seven components of the vector field f given by System (1). The vector field is quadratic with only four non-linear terms in x. Therefore, to compute *a*, it suffices to determine only the partial derivatives

$$\begin{array}{cc} \frac{\partial^{2}f_{1}}{\partial S\partial I_{j}} & =-\beta_{j},\;\frac{\partial^{2}f_{2}}{\partial V\partial I_{j}}=-\left(1-\phi \right)\beta_{j},\;\frac{\partial^{2}f_{3}}{\partial S\partial I_{j}}=\beta_{j},\;\mathrm{and}\;\;\frac{\partial^{2}f_{3}}{\partial V\partial I_{j}}=\left(1-\phi \right)\beta_{j} \end{array}$$

for j=1,2,3, along with their symmetric counterparts. All partial derivatives are evaluated at the DFE as well as at $\beta_{1}=\beta_{1}^{*}$. We then obtain

$$\begin{array}{cc} a & =2\left(\beta_{1}^{*}w_{4}+\beta_{2}w_{5}+\beta_{3}w_{6}\right)\left(v_{3}w_{1}+\left(1-\phi \right)v_{3}w_{2}-v_{1}w_{1}-\left(1-\phi \right)v_{2}w_{2}\right)\\ \; & =2v_{3}\left(\beta_{1}^{*}w_{4}+\beta_{2}w_{5}+\beta_{3}w_{6}\right)\left(w_{1}+\left(1-\phi \right)w_{2}\right)\\ \; & =2\frac{\mu \left(\mu +\epsilon \right)\left(\mu +p+\omega_{1}\right)}{\theta (\mu +\omega_{1}+p\left(1-\phi \right))}\left(w_{1}+\left(1-\phi \right)w_{2}\right). \end{array}$$

Since all parameters in *a* (including 1−ϕ, which is a probability) are positive, the sign of *a* depends on that of the factor $w_{1}+\left(1-\phi \right)w_{2}$, where $w_{1}$ and $w_{2}$ are aggregate parameters with indeterminate signs. In addition, the non-zero partial derivatives required for *b* are

$$\begin{array}{cc} \frac{\partial^{2}f_{1}}{\partial I_{1}\partial \beta_{1}} & =-S_{0},\;\frac{\partial^{2}f_{2}}{\partial I_{1}\partial \beta_{1}}=-\left(1-\phi \right)V_{0},\;\mathrm{and}\;\;\frac{\partial^{2}f_{3}}{\partial I_{1}\partial \beta_{1}}=S_{0}+\left(1-\phi \right)V_{0}. \end{array}$$

We then obtain

$$\begin{array}{cc} b & =v_{3}w_{4}\frac{\partial^{2}f_{3}}{\partial I_{1}\partial \beta_{1}}\\ \; & =v_{3}w_{4}\left(S_{0}+1-\phi \right)V_{0})\\ \; & =\frac{\theta \epsilon (\mu +\omega_{1}+p\left(1-\phi \right))}{\mu \left(\mu +p+\omega_{1}\right)L_{1}}>0. \end{array}$$

Since b>0, by Theorem 4.1 of the study by Castillo-Chavez and Song [31], it follows that the model undergoes a backward bifurcation at $R_{v}=1$, if a>0 (i.e., $w_{1}+\left(1-\phi \right)w_{2}>0$), which is equivalent to Condition (A9). □

**Remark A4.** 
*An investigation of Condition (A9) shows that there is no backward bifurcation in Model (1), when at least one of the following conditions holds: p=0 (i.e., there is no vaccination); ϕ=0 (i.e., the vaccine is completely ineffective); ϕ=1 (i.e., the vaccine induces perfect immunity); $\omega_{2}=0$ (i.e., the disease-acquired immunity does not wane); and μ=0 (i.e., natural mortality is excluded from the model). If the disease-acquired immunity does not wane ($\omega_{2}=0$), a leaky vaccine with waning immunity ($\omega_{1}\neq 0$) does not cause a backward bifurcation in the model. In addition, given the condition $\left(\mu +\epsilon -\omega_{2}\Lambda_{2}\right){\left(\mu +p\left(1-\phi \right)\right)}^{2}<\mu p\phi \left(1-\phi \right)\left(\mu +\epsilon \right)$, a leaky vaccine with no waning immunity ($\omega_{1}=0$) causes a backward bifurcation, when there is waning of the disease-acquired immunity ($\omega_{2}\neq 0$). Thus, the waning of infection-acquired immunity ($\omega_{2}$) has a more pronounced effect on the occurrence of a backward bifurcation in the model than does the waning of vaccine-induced immunity ($\omega_{1}$).*
